# Supplementary material for: Tinkering Cis Motifs Jigsaw Puzzle Led to Root-Specific Drought-Inducible Novel Synthetic Promoters
Source: Int J Mol Sci. 2020 Feb 18;21(4):1357. doi: 10.3390/ijms21041357 (PMC7072871; doi:10.3390/ijms21041357)
Supplement: Supplementary file 1 [file ijms-21-01357-s001.zip › Supplementary Files/Supplementary Figure S1.pdf]

Jump to: Multiple Alignment Motif Tree Motif Matching
Input file: 72 motifs loaded
Settings: Metric=PCC, Alignment=SWU, Gap-open=1000, Gap-extend=1000, -nooverlapalign
Multiple Alignment=IR, Tree=UPGMA, Matching against: AGRIS
Note: All results files are removed nightly at midnight EST. Please save your results by saving "Webpage, complete".
Download results as a PDF
Click here to run STAMP again.

Multiple Alignment

(Consensus sequence representations shown, but multiple alignment was carried out on the matrices)

```
meme_cluster3_1:       -----------CRCCTCMACA-----------------
biop_10_cluster1_1:    -----------------CACGTGNC-------------
biop_10_cluster1_2:    -----------------CACGTGNC-------------
biop_10_cluster1_3:    ------------CCCYYCWYCC----------------
aa_10_cluster3_1:      -----------------WAAAAAAWAA-----------
aa_10_cluster3_2:      ------------------AAAAAWAANWWWAAWAWAWA
meme_cluster4_1:       ------------GNCSCCACNY----------------
meme_cluster4_2:       ------------RKNGMCACGTGKC-------------
meme_cluster4_3:       --------------CCCNACCC----------------
meme_cluster4_4:       ---------------GMCACGTGKCNNY----------
meme_cluster4_5:       ----------------MCACGTGKCNMCN---------
aa_10_cluster2_1:      ------------------AAAAAAAAAA----------
aa_10_cluster2_2:      -------TTWWWWWWNWAAAWWAWT-------------
aa_10_cluster2_3:      ------------AANAWAAAATWAAWWWAAAAAWAW--
aa_10_cluster2_4:      ----TWWWWWAWTTTTAAAAW-----------------
aa_10_cluster2_5:      ----------------MCACGTGK--------------
con_10_cluster4_1:     ------------GSNSCCACG-----------------
con_10_cluster4_2:     ------------RKNGMCACGTGKC-------------
con_10_cluster4_3:     ------------SGCSACACKNNNC-------------
con_10_cluster4_4:     ------------GSYGCCACGY----------------
con_10_cluster4_5:     -----------TGKCGMCACS-----------------
meme_cluster1_1:       ----------------YCWCTCNYCC------------
meme_cluster1_2:       ---------------GNCACGTGNC-------------
```

```
meme_cluster1_3:         ----------TYTYTYTCTC------------------
meme_cluster1_4:         --------------CCNCNCNNYC--------------
meme_cluster1_5:         ---------------NNCACGTG---------------
aa_10_all_cluster_1:     ---------AAAAAAAAWA-------------------
aa_10_all_cluster_2:     ---------AAAAAWNAAAW------------------
aa_10_all_cluster_3:     --------WWWWWWAAWTAATTWWWW------------
aa_10_all_cluster_4:     -----------AAAAATAAAAATAAW------------
aa_10_all_cluster_5:     WWWWTTWWWWWWWWWAAAA-------------------
con_10_cluster1_1:       -----------------CACGTGNN-------------
con_10_cluster1_2:       ---------------NNCACGTGNC-------------
con_10_cluster1_3:       -------------CCCWCMCS-----------------
con_10_cluster1_4:       -----------CNCNCYYNCNY----------------
con_10_cluster1_5:       -----------------CACGTG---------------
biop_10_all_cluster_:    --------CCYNNCNNNCNCC-----------------
biop_10_all_clust_v2:    --------CCYYYCNYYCNC------------------
biop_10_all_clust_v3:    -----------------CACGTG---------------
biop_10_all_clust_v4:    ----------------MCACGTG---------------
meme_cluster2_1:         ----------------MCACGTGK--------------
meme_cluster2_2:         -----------GGGNSNSRCC-----------------
meme_cluster2_3:         -----------------CACSCGCCSM-----------
meme_cluster2_4:         ------------TYNCTCTCTC----------------
meme_cluster2_5:         ---------------RMCACGTGK--------------
aa_10_cluster4_1:        -------AAWWWWWWWAAAAANWWA-------------
aa_10_cluster4_2:        -----ANNWNWAAAANAWANW-----------------
aa_10_cluster4_3:        -----------TWWTAAAAWWW----------------
aa_10_cluster4_4:        ---AAWWAWWWWWWWWWAAWAW----------------
aa_10_cluster4_5:        ------------RKNGMCACGTGK--------------
meme_all_cluster_1:      ----------------MCACGTG---------------
meme_all_cluster_2:      -----------------CNCTYNYCCY-----------
meme_all_cluster_3:      -----------------CACGTG---------------
meme_all_cluster_4:      -----------------CACGTG---------------
meme_all_cluster_5:      ---------AAAAAAAAAA-------------------
biop_10_cluster2_1:      -----------------CNCWCCMYC------------
biop_10_cluster2_2:      -----------------CWCSYSNCCYC----------
biop_10_cluster2_3:      -------------RNSMCACS-----------------
biop_10_cluster2_4:      -----------------CACGTG---------------
biop_10_cluster3_1:      ----------------GCAYSGYNSA------------
biop_10_cluster4_1:      ------------RKNGMCACGTGKC-------------
biop_10_cluster4_2:      ------------RKNGMCACGTGKC-------------
biop_10_cluster4_3:      ------------GSNGMCACGT----------------
biop_10_cluster4_4:      ---------------GMCACGTGNCNNY----------
biop_10_cluster4_5:      -------------NCNMCNCGTSNC-------------
con_10_cluster3_1:       --------------RNGCANGGTGSN------------
```

```
con_10_cluster3_2:    ---------------NGCACGKTGCG------------
aa_10_cluster1_1:     ----------------AAAAAAANAAW-----------
aa_10_cluster1_2:     ---TTTTTTTTTTTMWNW-------------------
aa_10_cluster1_3:     ---------------GNCACGTGNC-------------
aa_10_cluster1_4:     -------------YYCCMNCNCNCNYYC----------
aa_10_cluster1_5:     ------------TCNCNNNCNCNNCCNNNC--------
```

Familial Profile:
(click for matrix)

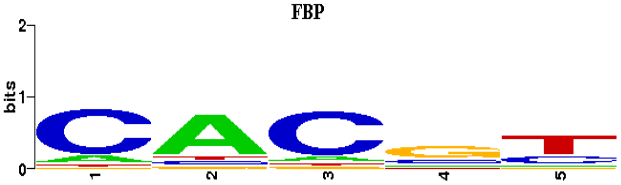

Motif Tree

Tree (drawn by Phylip)
Click here for Newick-format tree (viewable with MEGA)

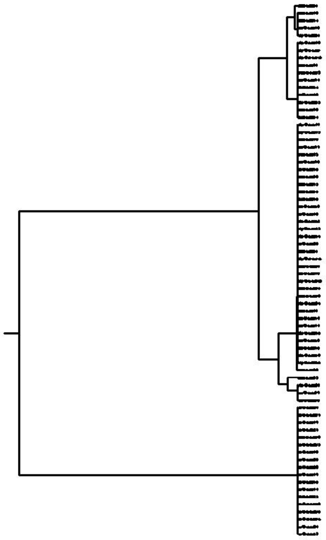

Input Motif	Best match in AGRIS
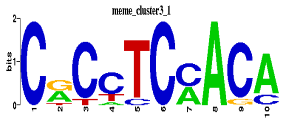 meme_cluster3_1	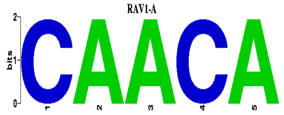 RAV1-A (E val: 4.2977e-04)
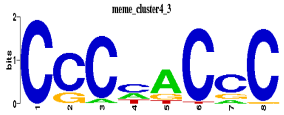 meme_cluster4_3	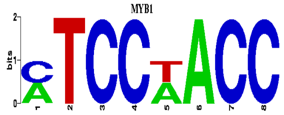 MYB1 (E val: 6.5555e-04)
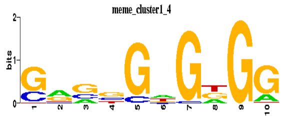 meme_cluster1_4	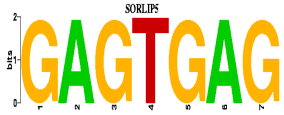 SORLIP5 (E val: 1.8114e-04)
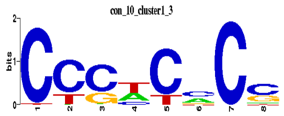 con_10_cluster1_3	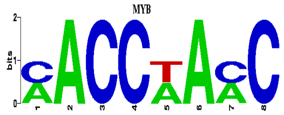 MYB (E val: 1.1384e-02)

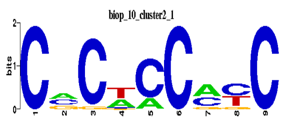<br>biop_10_cluster2_1	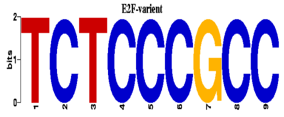<br>E2F-varient<br>(E val: 1.3407e-02)
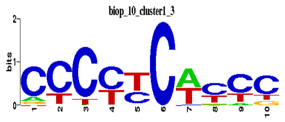<br>biop_10_cluster1_3	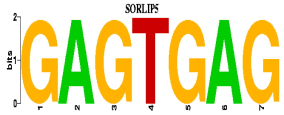<br>SORLIP5<br>(E val: 3.9003e-04)
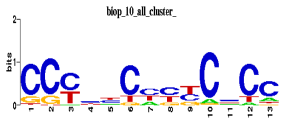<br>biop_10_all_cluster_	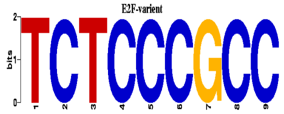<br>E2F-varient<br>(E val: 1.0524e-03)
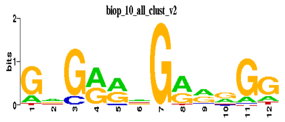<br>biop_10_all_clust_v2	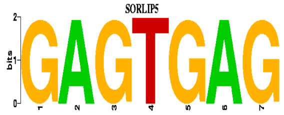<br>SORLIP5<br>(E val: 7.4007e-03)
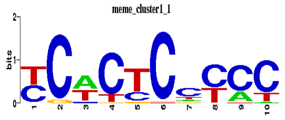<br>meme_cluster1_1	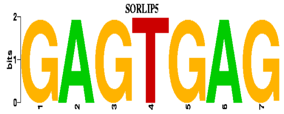<br>SORLIP5<br>(E val: 2.3050e-04)
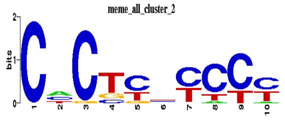<br>meme_all_cluster_2	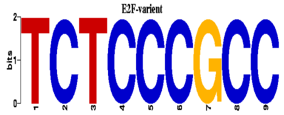<br>E2F-varient<br>(E val: 2.3243e-03)
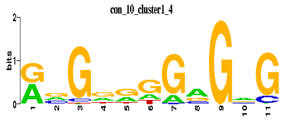<br>con_10_cluster1_4	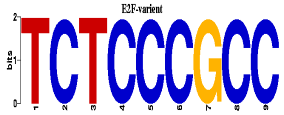<br>E2F-varient<br>(E val: 4.7094e-05)
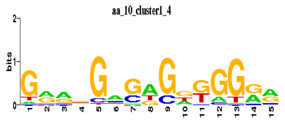<br>aa_10_cluster1_4	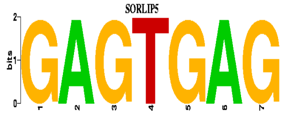<br>SORLIP5<br>(E val: 2.6287e-03)

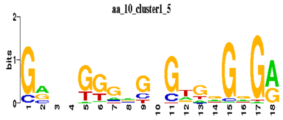<br>aa_10_cluster1_5	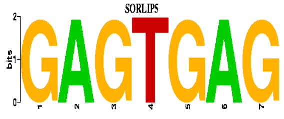<br>SORLIP5<br>(E val: 3.9470e-05)
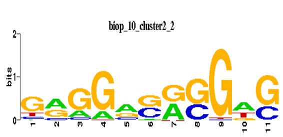<br>biop_10_cluster2_2	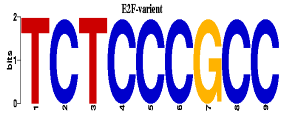<br>E2F-varient<br>(E val: 1.3850e-03)
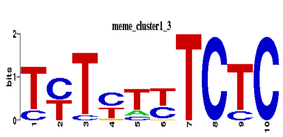<br>meme_cluster1_3	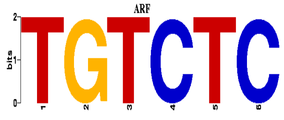<br>ARF<br>(E val: 1.7015e-03)
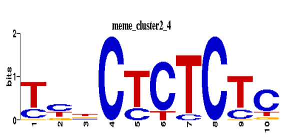<br>meme_cluster2_4	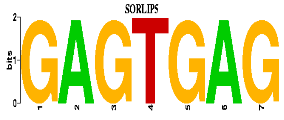<br>SORLIP5<br>(E val: 4.8964e-05)
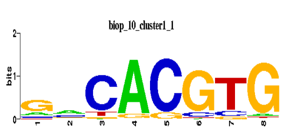<br>biop_10_cluster1_1	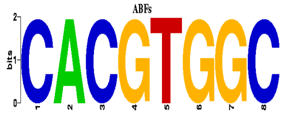<br>ABFs<br>(E val: 4.9218e-11)
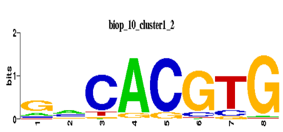<br>biop_10_cluster1_2	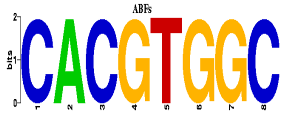<br>ABFs<br>(E val: 3.2649e-11)
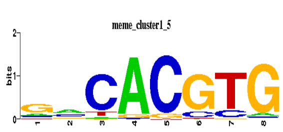<br>meme_cluster1_5	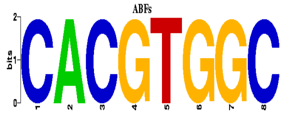<br>ABFs<br>(E val: 1.0318e-11)
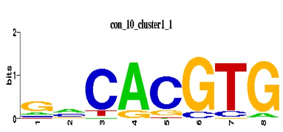<br>con_10_cluster1_1	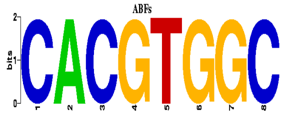<br>ABFs<br>(E val: 6.1569e-12)

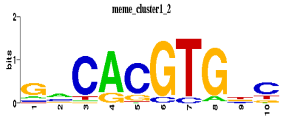 meme_cluster1_2	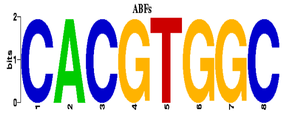 ABFs (E val: 4.9057e-10)
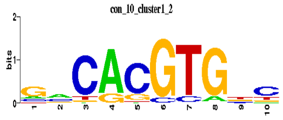 con_10_cluster1_2	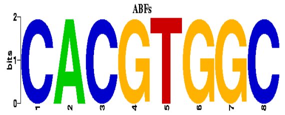 ABFs (E val: 3.5788e-10)
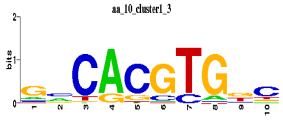 aa_10_cluster1_3	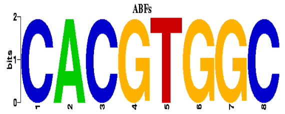 ABFs (E val: 1.2256e-10)
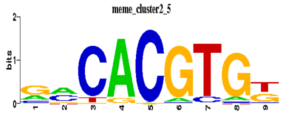 meme_cluster2_5	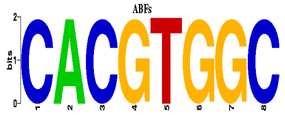 ABFs (E val: 3.6560e-11)
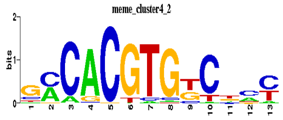 meme_cluster4_2	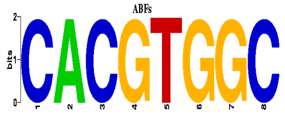 ABFs (E val: 4.5627e-10)
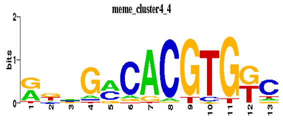 meme_cluster4_4	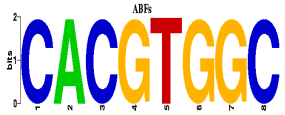 ABFs (E val: 2.9884e-10)
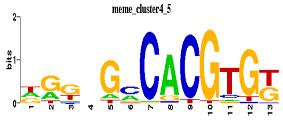 meme_cluster4_5	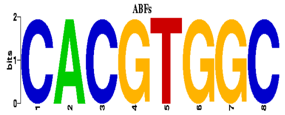 ABFs (E val: 8.9105e-11)
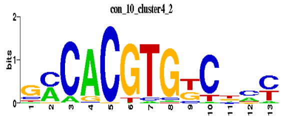 con_10_cluster4_2	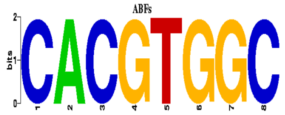 ABFs (E val: 4.5627e-10)

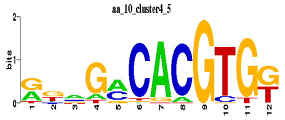 aa_10_cluster4_5	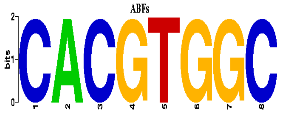 ABFs (E val: 2.7376e-09)
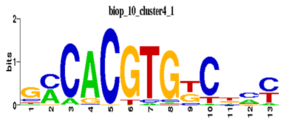 biop_10_cluster4_1	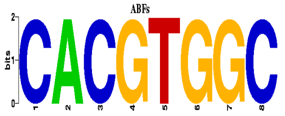 ABFs (E val: 4.5627e-10)
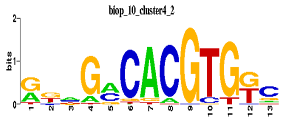 biop_10_cluster4_2	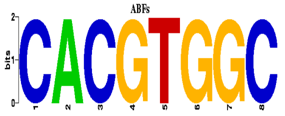 ABFs (E val: 4.5627e-10)
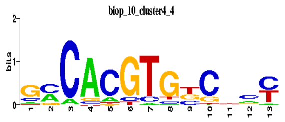 biop_10_cluster4_4	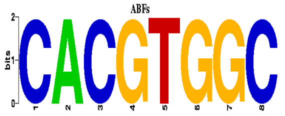 ABFs (E val: 3.9958e-10)
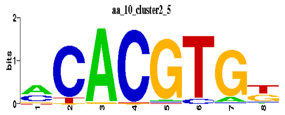 aa_10_cluster2_5	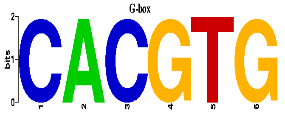 G-box (E val: 4.2821e-09)
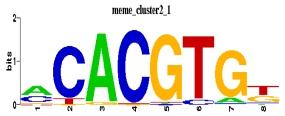 meme_cluster2_1	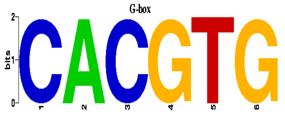 G-box (E val: 4.3084e-09)
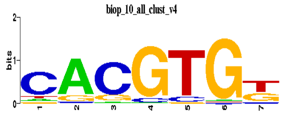 biop_10_all_clust_v4	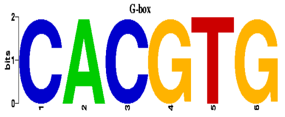 G-box (E val: 1.2324e-09)
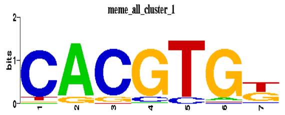 meme_all_cluster_1	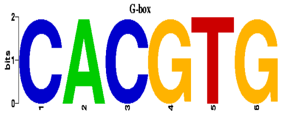 G-box (E val: 1.1774e-09)

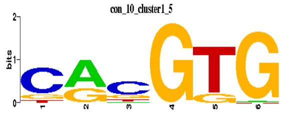 con_10_cluster1_5	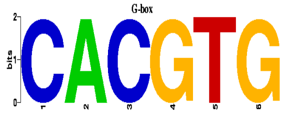 G-box (E val: 4.5925e-10)
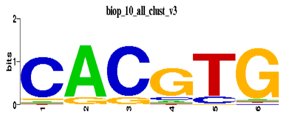 biop_10_all_clust_v3	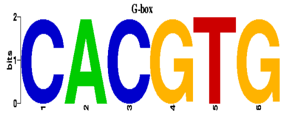 G-box (E val: 6.2438e-11)
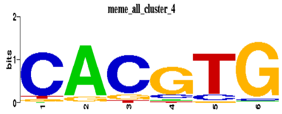 meme_all_cluster_4	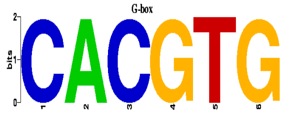 G-box (E val: 5.4745e-11)
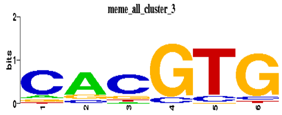 meme_all_cluster_3	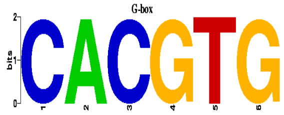 G-box (E val: 5.9453e-11)
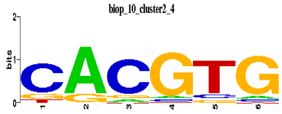 biop_10_cluster2_4	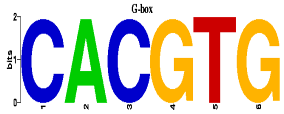 G-box (E val: 6.9934e-11)
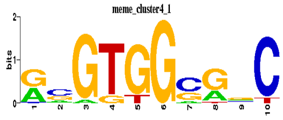 meme_cluster4_1	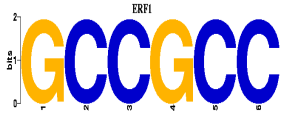 ERF1 (E val: 3.7384e-07)
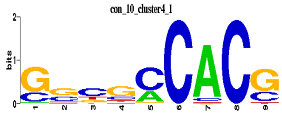 con_10_cluster4_1	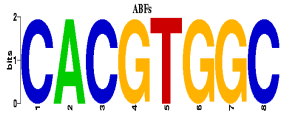 ABFs (E val: 2.5416e-05)
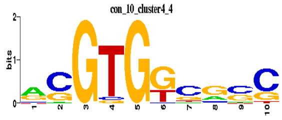 con_10_cluster4_4	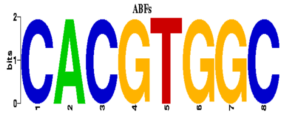 ABFs (E val: 1.2568e-07)

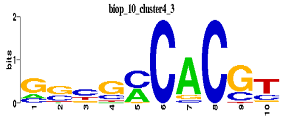 biop_10_cluster4_3	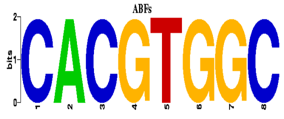 ABFs (E val: 2.0624e-07)
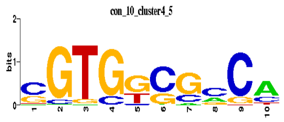 con_10_cluster4_5	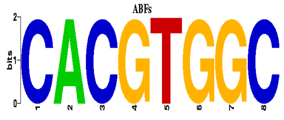 ABFs (E val: 1.3922e-04)
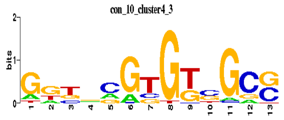 con_10_cluster4_3	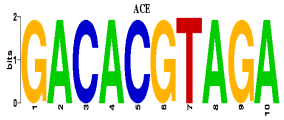 ACE (E val: 6.2490e-05)
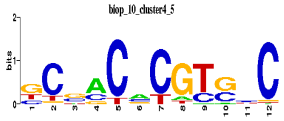 biop_10_cluster4_5	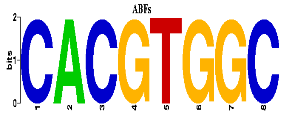 ABFs (E val: 2.9123e-06)
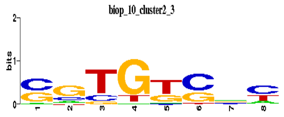 biop_10_cluster2_3	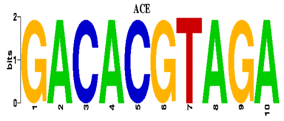 ACE (E val: 6.1809e-04)
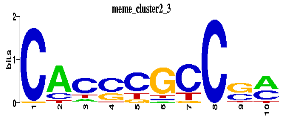 meme_cluster2_3	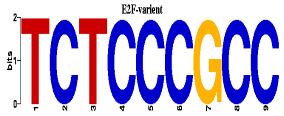 E2F-varient (E val: 3.9617e-06)
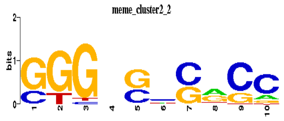 meme_cluster2_2	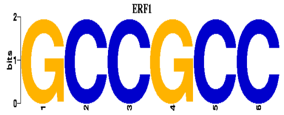 ERF1 (E val: 1.3269e-04)
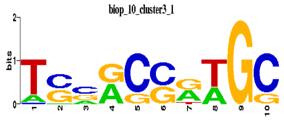 biop_10_cluster3_1	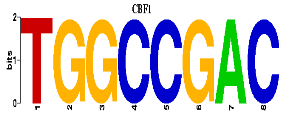 CBF1 (E val: 8.9174e-03)

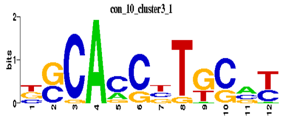 con_10_cluster3_1	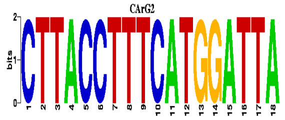 CArG2<br>(E val: 1.7770e-03)
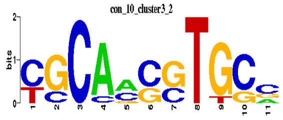 con_10_cluster3_2	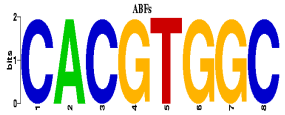 ABFs<br>(E val: 1.3651e-05)
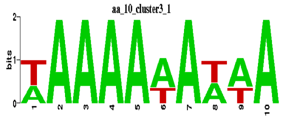 aa_10_cluster3_1	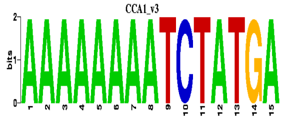 CCA1_v3<br>(E val: 6.6562e-06)
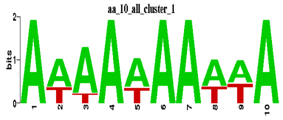 aa_10_all_cluster_1	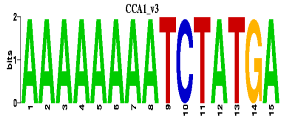 CCA1_v3<br>(E val: 7.1473e-08)
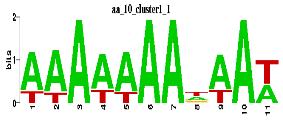 aa_10_cluster1_1	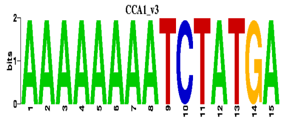 CCA1_v3<br>(E val: 9.5610e-07)
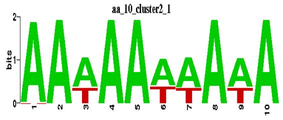 aa_10_cluster2_1	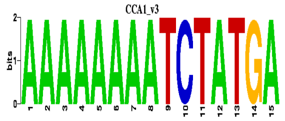 CCA1_v3<br>(E val: 2.7265e-07)
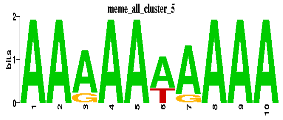 meme_all_cluster_5	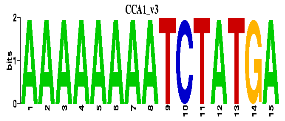 CCA1_v3<br>(E val: 1.3989e-07)
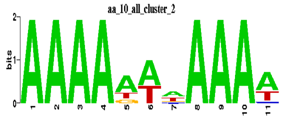 aa_10_all_cluster_2	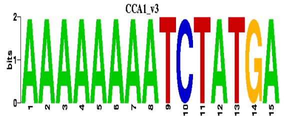 CCA1_v3<br>(E val: 3.6212e-06)

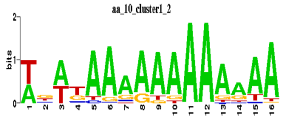 aa_10_cluster1_2	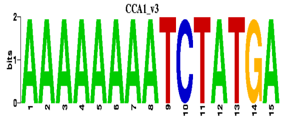 CCA1_v3 (E val: 1.8433e-04)
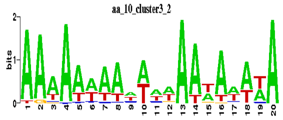 aa_10_cluster3_2	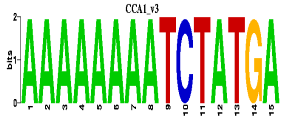 CCA1_v3 (E val: 6.8505e-06)
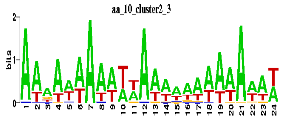 aa_10_cluster2_3	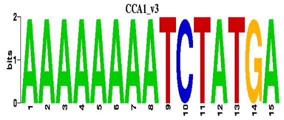 CCA1_v3 (E val: 2.1018e-05)
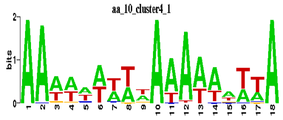 aa_10_cluster4_1	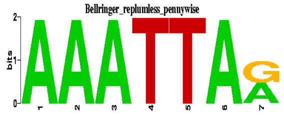 Bellringer_replumless_pennywise (E val: 9.2666e-04)
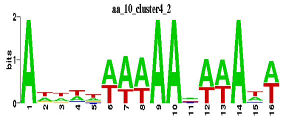 aa_10_cluster4_2	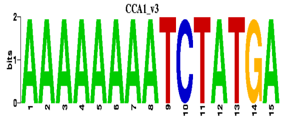 CCA1_v3 (E val: 2.3128e-04)
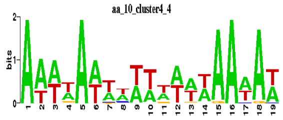 aa_10_cluster4_4	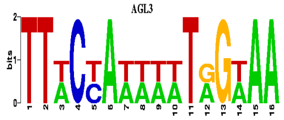 AGL3 (E val: 3.9512e-05)
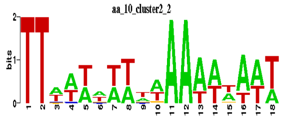 aa_10_cluster2_2	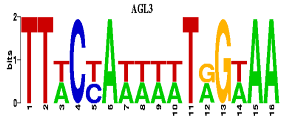 AGL3 (E val: 2.9105e-06)
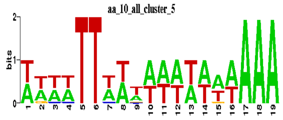 aa_10_all_cluster_5	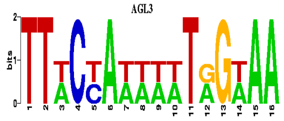 AGL3 (E val: 1.6908e-06)

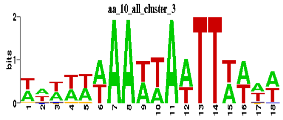 aa_10_all_cluster_3	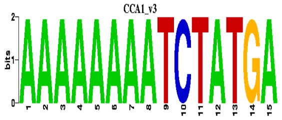 CCA1_v3 (E val: 8.7808e-05)
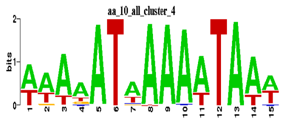 aa_10_all_cluster_4	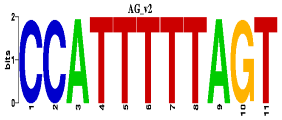 AG_v2 (E val: 8.1216e-06)
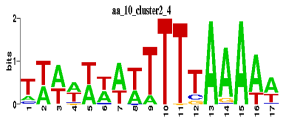 aa_10_cluster2_4	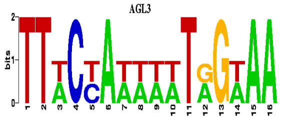 AGL3 (E val: 4.3648e-06)
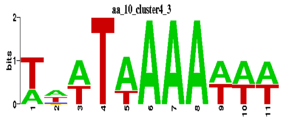 aa_10_cluster4_3	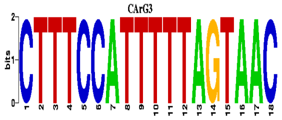 CArG3 (E val: 3.1400e-04)

Motif Similarity Matches

meme_cluster3_1

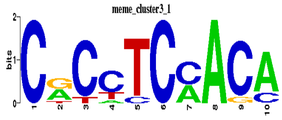

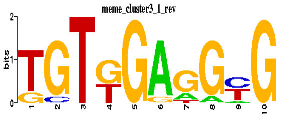

forward *reverse compliment*

Name	*E value*	*Alignment*	*Motif*
RAV1-A	4.2977e-04	CRCCTCMACA<br>------CAACA	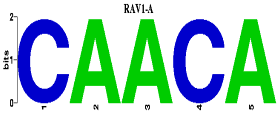
RAV1-B	1.3312e-03	TGTKGAGGYG<br>----CAGGTG	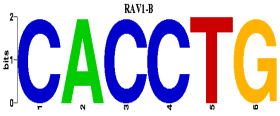
TGA1	2.5764e-02	-CRCCTCMACA<br>CCACGTCA---	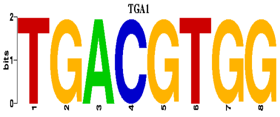

ERF1	2.9515e-02	TGTKGAGGYG<br>---GGCGGC-	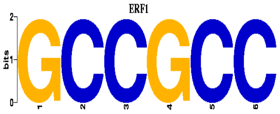
GCC-box	2.9515e-02	TGTKGAGGYG<br>---GGCGGC-	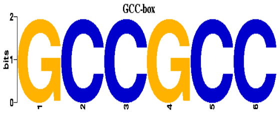

---

meme_cluster4_3

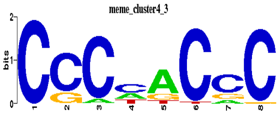

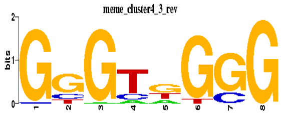

forward *reverse compliment*

Name	*E value*	*Alignment*	*Motif*
MYB1	6.5555e-04	GGGTNGGG-<br>-GGTWGGAK	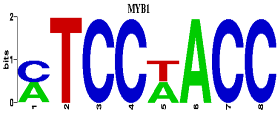
MYB	5.2232e-03	GGGTNGGG-<br>-GKTWGGTK	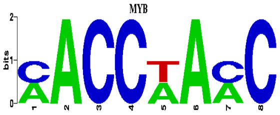
E2F-varient	1.1996e-02	GGGTNGGG--<br>-GGCGGGAGA	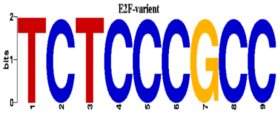
MRE	1.3447e-02	GGGTNGGG----<br>TGGTAGGTTAGA	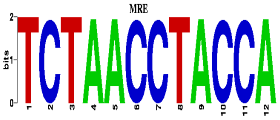
MYB4	1.8585e-02	CCCNACCC<br>AMCWAMC-	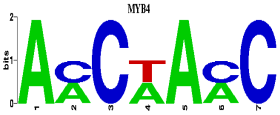

---

meme_cluster1_4

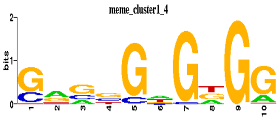

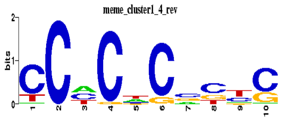

forward *reverse compliment*

Name	*E value*	*Alignment*	*Motif*

SORLIP5	1.8114e-04	GRNNGNGNGG<br>GAGTGAG---	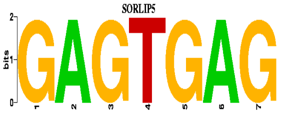
E2F-varient	3.9895e-02	-GRNNGNGNGG<br>GGCGGGAGA--	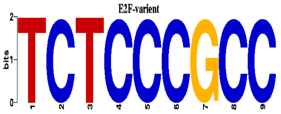
MYB4	6.2511e-02	GRNNGNGNGG<br>--GKTWGKT-	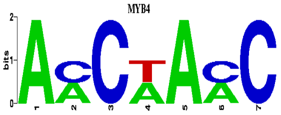
GT	7.6462e-02	--------CCNCNCNNYC<br>CATATTAACCACACA---	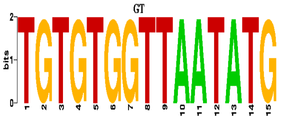
SORLIP1	9.1186e-02	--CCNCNCNNYC<br>AGCCAC------	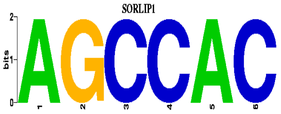

---

con_10_cluster1_3

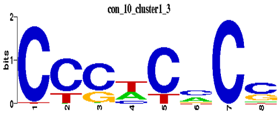

forward

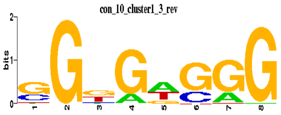

reverse compliment

Name	*E value*	*Alignment*	*Motif*
MYB	1.1384e-02	SGKGWGGG-<br>-GKTWGGTK	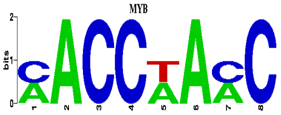
SORLIP5	1.2978e-02	CCCWCMCS-<br>--CTCACTC	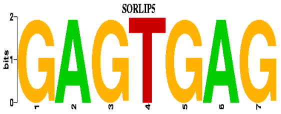
Hexamer	2.1896e-02	SGKGWGGG<br>--CGACGG	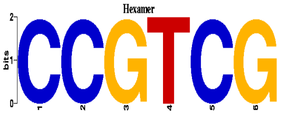
E2F-varient	2.4054e-02	--SGKGWGGG<br>GGCGGGAGA-	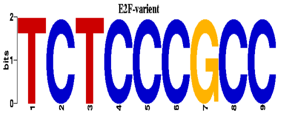

LTRE	2.8405e-02	SGKGWGGG-<br>--TGTCGGT	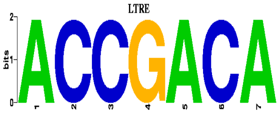

biop_10_cluster2_1

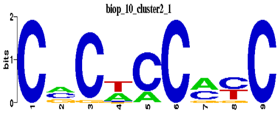

forward

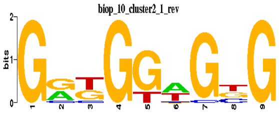

reverse compliment

Name	*E value*	*Alignment*	*Motif*
E2F-varient	1.3407e-02	-GRKGGWGNG<br>GGCGGGAGA-	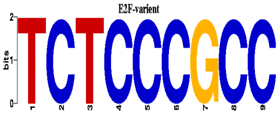
SORLIP5	1.4550e-02	--CNCWCCMYC<br>CTCACTC----	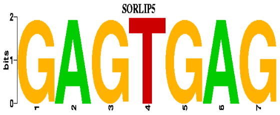
RAV1-B	8.3237e-02	--GRKGGWGNG<br>CAGGTG-----	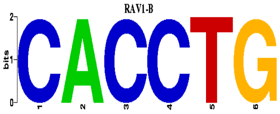
SORLIP4	8.8920e-02	CNCWCCMYC----<br>----CCATCATAC	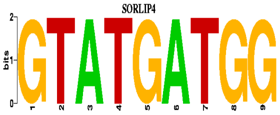
SORLIP1	9.4131e-02	CNCWCCMYC<br>--AGCCAC-	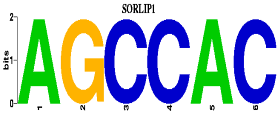

biop_10_cluster1_3

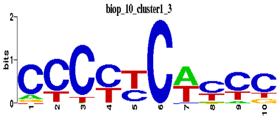

forward

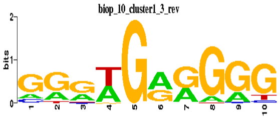

reverse compliment

Name	*E value*	*Alignment*	*Motif*

SORLIP5	3.9003e-04	GGRWGRRGGG<br>GAGTGAG---	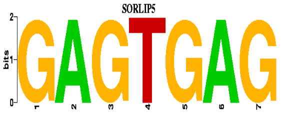
E2F-varient	7.2098e-03	---GGRWGRRGGG<br>GGCGGGAGA----	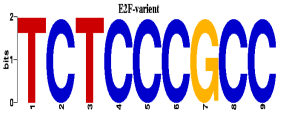
MYB1	1.5792e-02	GGRWGRRGGG<br>GGTWGGAK--	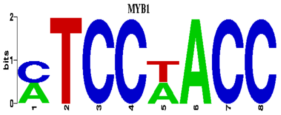
SORLIP4	3.4084e-02	CCCYYCWYCC<br>-CCATCATAC	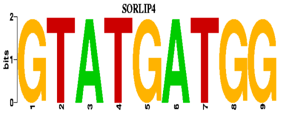
RAV1-B	1.0313e-01	CCCYYCWYCC-<br>-----CACCTG	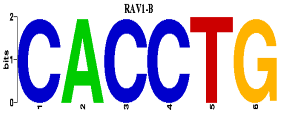

---

biop_10_all_cluster_

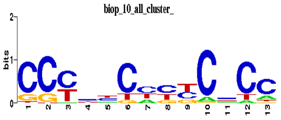

forward

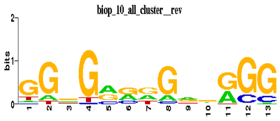

reverse compliment

Name	*E value*	*Alignment*	*Motif*
E2F-varient	1.0524e-03	GGNGNNNGNNRGG<br>--GGCGGGAGA--	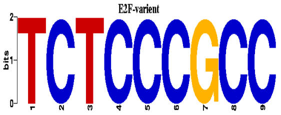
SORLIP5	1.9620e-03	CCYNNCNNNCNCC<br>---CTCACTC---	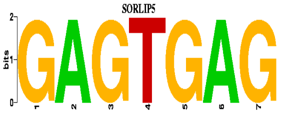
TEF-box	6.2993e-02	-GGNGNNNGNNRGG--<br>AGGGGCATAATGGTAA	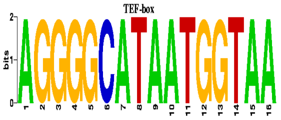
E2F	1.5702e-01	-GGNGNNNGNNRGG<br>GCGGGAAA------	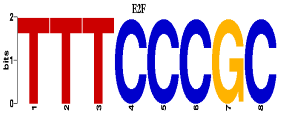

E2F_DP	1.5702e-01	-GGNGNNNGNNRGG<br>GCGGGAAA------	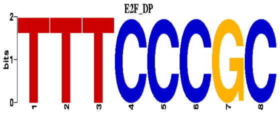

biop_10_all_clust_v2

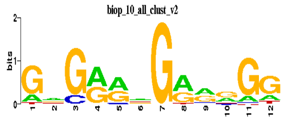

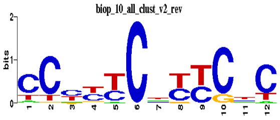

forward *reverse compliment*

Name	*E value*	*Alignment*	*Motif*
SORLIP5	7.4007e-03	CCYYYCNYYCNC<br>---CTCACTC--	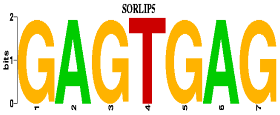
E2F-varient	4.5482e-02	-GNGRRNGRRRGG<br>GGCGGGAGA----	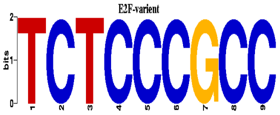
ARF	7.2518e-02	GNGRRNGRRRGG<br>GAGACA------	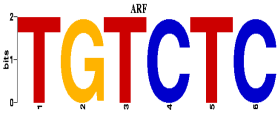
ARF1	7.2518e-02	GNGRRNGRRRGG<br>GAGACA------	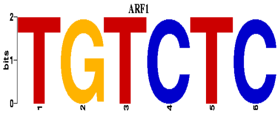
CArG2	9.4405e-02	--GNGRRNGRRRGG----<br>TAATCCATGAAAGGTAAG	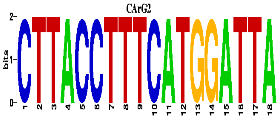

meme_cluster1_1

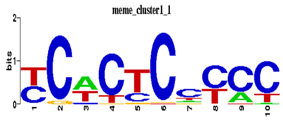

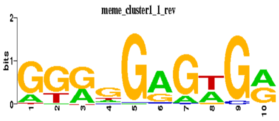

forward *reverse compliment*

Name	*E value*	*Alignment*	*Motif*

SORLIP5	2.3050e-04	-YCWCTCNYCC<br>CTCACTC----	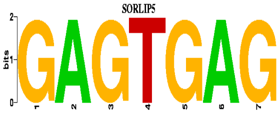
E2F-varient	6.8582e-04	YCWCTCNYCC-<br>--TCTCCCGCC	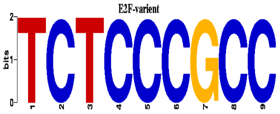
EIL1	2.1501e-02	---------YCWCTCNYCC-----<br>TTCAAGATACATGCCCCCTTGAA	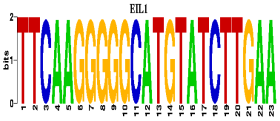
EIL2	2.1501e-02	---------YCWCTCNYCC-----<br>TTCAAGATACATGCCCCCTTGAA	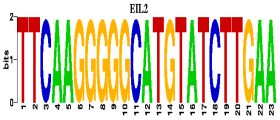
EIL3	2.1501e-02	---------YCWCTCNYCC-----<br>TTCAAGATACATGCCCCCTTGAA	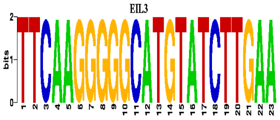

---

meme_all_cluster_2

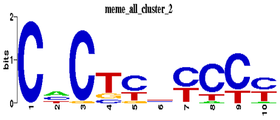

forward

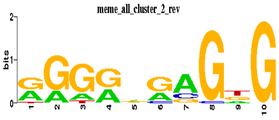

reverse compliment

Name	*E value*	*Alignment*	*Motif*
E2F-varient	2.3243e-03	RGGRNRAGNG<br>GGCGGGAGA-	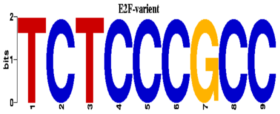
SORLIP5	5.7760e-03	RGGRNRAGNG--<br>-----GAGTGAG	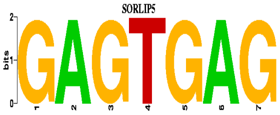
EIL1	2.1809e-02	---------CNCTYNYCCY----<br>TTCAAGATACATGCCCCCTTGAA	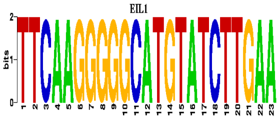
EIL2	2.1809e-02	---------CNCTYNYCCY----<br>TTCAAGATACATGCCCCCTTGAA	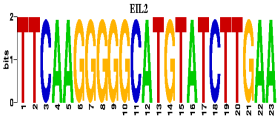
EIL3	2.1809e-02	---------CNCTYNYCCY----<br>TTCAAGATACATGCCCCCTTGAA	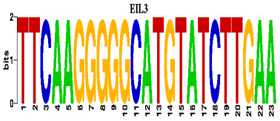

con_10_cluster1_4

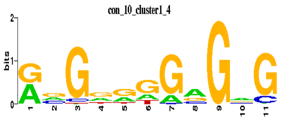

forward

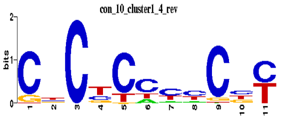

reverse compliment

Name	*E value*	*Alignment*	*Motif*
E2F-varient	4.7094e-05	RNGNRRGNGNG<br>-GGCGGGAGA-	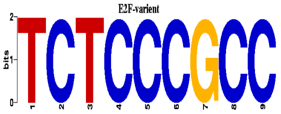
SORLIP5	2.2573e-03	RNGNRRGNGNG<br>--GAGTGAG--	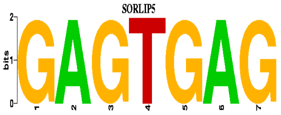
MYB1	3.1129e-02	RNGNRRGNGNG<br>-GGTWGGAK--	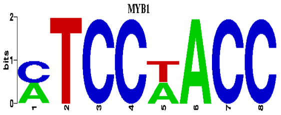
E2F	3.9542e-02	CNCNCYYNCNY<br>-TTTCCCGC--	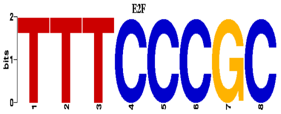
E2F_DP	3.9542e-02	CNCNCYYNCNY<br>-TTTCCCGC--	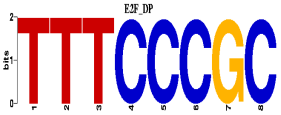

aa_10_cluster1_4

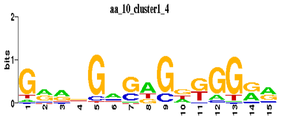

forward

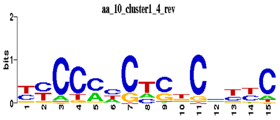

reverse compliment

Name	*E value*	*Alignment*	*Motif*
SORLIP5	2.6287e-03	YYCCMNCNCNCNYYC<br>--------CTCACTC	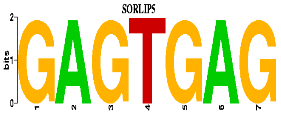

E2F-varient	1.8220e-02	-GRRNGNGNGNKGGRR<br>GGCGGGAGA-------	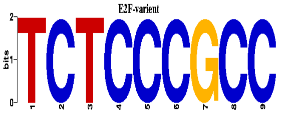
EIN3	2.2770e-02	GRRNGNGNGNKGGRR--------------<br>GGATTCAAGGGGGCATGTATCTTGAATCC	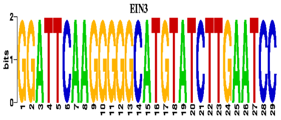
ATB2_AtbZIP53_AtbZIP44_GBF5	5.8095e-02	GRRNGNGNGNKGGRR<br>--ATGAGT-------	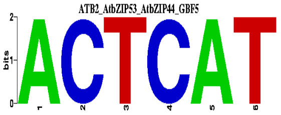
TGA1	6.0368e-02	YYCCMNCNCNCNYYC<br>--CCACGTCA-----	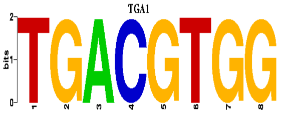

aa_10_cluster1_5

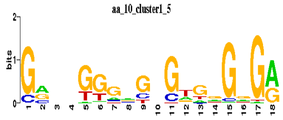

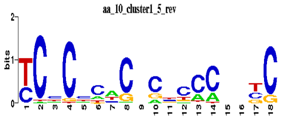

forward *reverse compliment*

Name	*E value*	*Alignment*	*Motif*
SORLIP5	3.9470e-05	TCNCNNNCNCNNCCNNNC<br>---CTCACTC--------	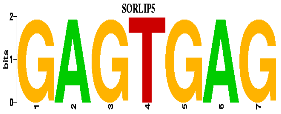
E2F-varient	1.5414e-02	GNNNGGNNGNGNNNGNGA<br>-GGCGGGAGA--------	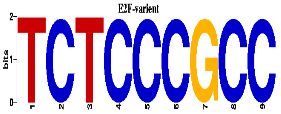
TEF-box	2.9816e-02	TCNCNNNCNCNNCCNNNC<br>-TTACCATTATGCCCCT-	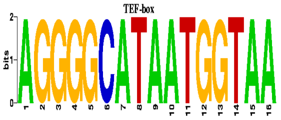
ATB2_AtbZIP53_AtbZIP44_GBF5	1.7322e-01	TCNCNNNCNCNNCCNNNC<br>------ACTCAT------	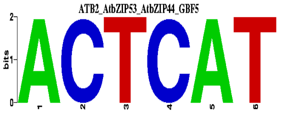
SORLIP1	1.8318e-01	GNNNGGNNGNGNNNGNGA<br>----------GTGGCT--	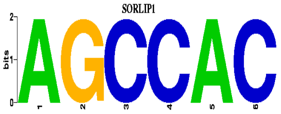

biop_10_cluster2_2

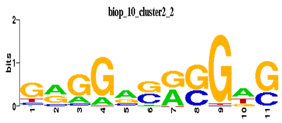

forward

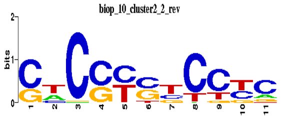

reverse compliment

Name	*E value*	*Alignment*	*Motif*
E2F-varient	1.3850e-03	GRGGNSRSGWG-<br>---GGCGGGAGA	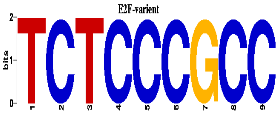
RAV1-B	2.4525e-02	GRGGNSRSGWG<br>-----CAGGTG	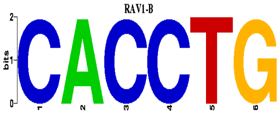
Hexamer	4.9736e-02	GRGGNSRSGWG<br>--CGACGG---	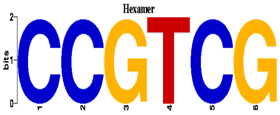
ABFs	5.0832e-02	GRGGNSRSGWG<br>---GCCACGTG	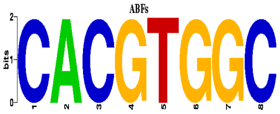
ABRE-like	5.9108e-02	GRGGNSRSGWG<br>---KMCACGTN	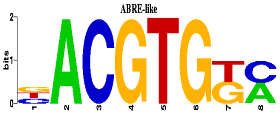

---

meme_cluster1_3

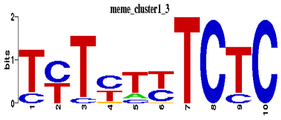

forward

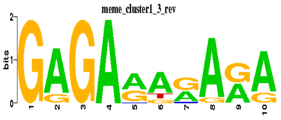

reverse compliment

Name	*E value*	*Alignment*	*Motif*
ARF	1.7015e-03	GAGARARARA<br>GAGACA----	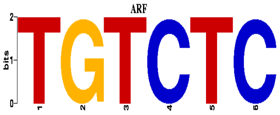
ARF1	1.7015e-03	GAGARARARA<br>GAGACA----	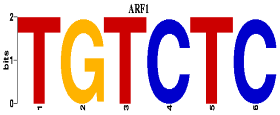

SORLIP5	3.0207e-03	GAGARARARA<br>GAGTGAG---	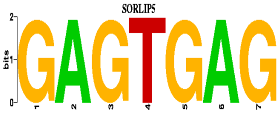
CCA1_v3	1.2274e-02	-------TYTYTYTCTC<br>TCATAGATTTTTTTT--	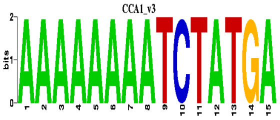
E2F	1.4138e-02	--GAGARARARA<br>GCGGGAAA----	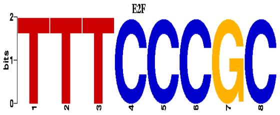

meme_cluster2_4

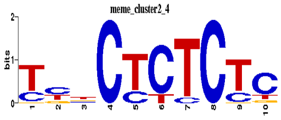

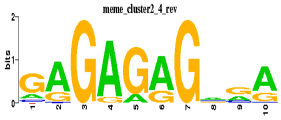

forward *reverse compliment*

Name	*E value*	*Alignment*	*Motif*
SORLIP5	4.8964e-05	TYNCTCTCTC<br>---CTCACTC	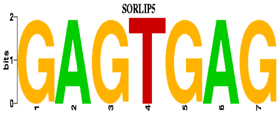
ARF	1.7638e-03	GAGAGAGNRA<br>--GAGACA--	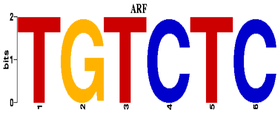
ARF1	1.7638e-03	GAGAGAGNRA<br>--GAGACA--	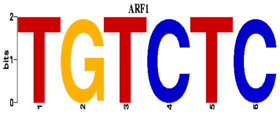
E2F-varient	2.9570e-03	TYNCTCTCTC<br>TCTCCCGCC-	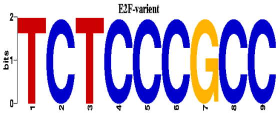
E2F	1.0061e-02	GAGAGAGNRA<br>--GCGGGAAA	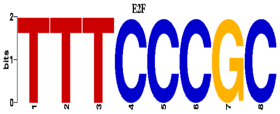

biop_10_cluster1_1

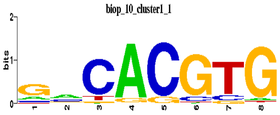

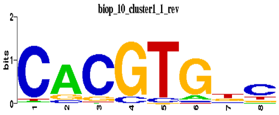

forward *reverse compliment*

Name	*E value*	*Alignment*	*Motif*
ABFs	4.9218e-11	GNCACGTG<br>GCCACGTG	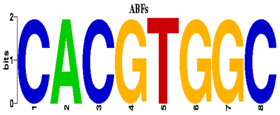
ABRE	2.1092e-09	GNCACGTG<br>GCCACGTR	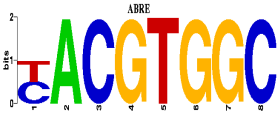
G-box	5.2985e-09	GNCACGTG<br>--CACGTG	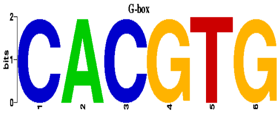
ABRE-like	8.1440e-09	GNCACGTG<br>KMCACGTN	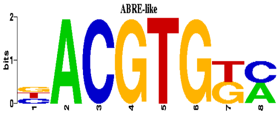
ACE	5.2527e-08	GNCACGTG--<br>GACACGTAGA	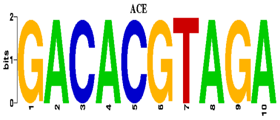

biop_10_cluster1_2 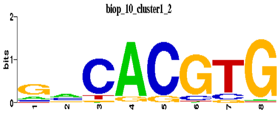

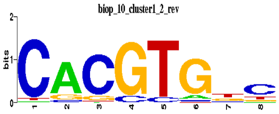

forward *reverse compliment*

Name	*E value*	*Alignment*	*Motif*
ABFs	3.2649e-11	CACGTGNC<br>CACGTGGC	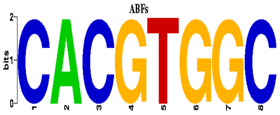
ABRE	1.5362e-09	CACGTGNC<br>YACGTGGC	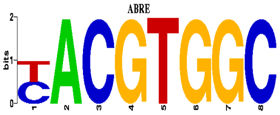
G-box	5.3569e-09	CACGTGNC<br>CACGTG--	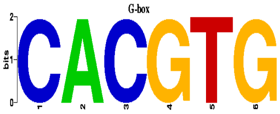

ABRE-like	7.4835e-09	CACGTGNC<br>NACGTGKM	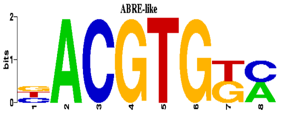
ACE	6.8080e-08	--CACGTGNC<br>TCTACGTGTC	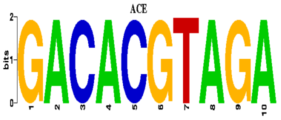

---

meme_cluster1_5

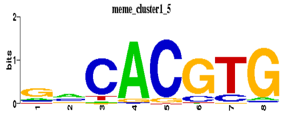

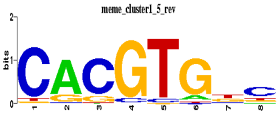

forward *reverse compliment*

Name	*E value*	*Alignment*	*Motif*
ABFs	1.0318e-11	NNCACGTG<br>GCCACGTG	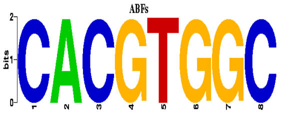
ABRE	4.5077e-10	NNCACGTG<br>GCCACGTR	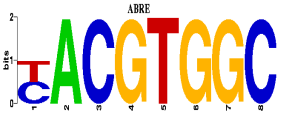
ABRE-like	2.6600e-09	NNCACGTG<br>KMCACGTN	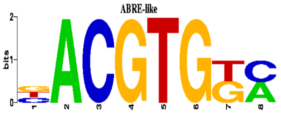
G-box	5.7685e-09	NNCACGTG<br>--CACGTG	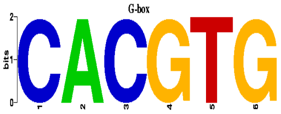
CBF2	8.4437e-08	NNCACGTG-<br>-CCACGTGG	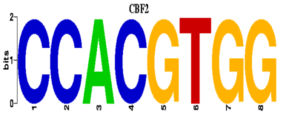

---

con_10_cluster1_1

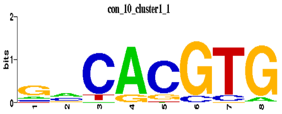

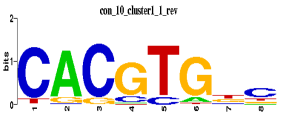

forward *reverse compliment*

Name	*E value*	*Alignment*	*Motif*
ABFs	6.1569e-12	NNCACGTG<br>GCCACGTG	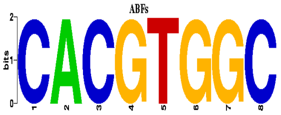
ABRE	1.9523e-10	NNCACGTG<br>GCCACGTR	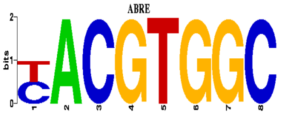
ABRE-like	3.2575e-09	NNCACGTG<br>KMCACGTN	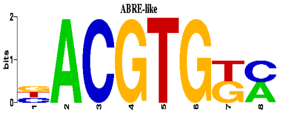
G-box	6.3426e-09	NNCACGTG<br>--CACGTG	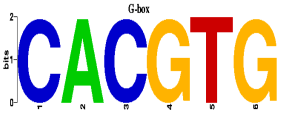
CBF2	5.5909e-08	NNCACGTG-<br>-CCACGTGG	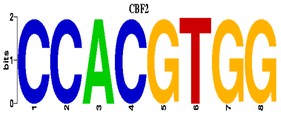

---

meme_cluster1_2

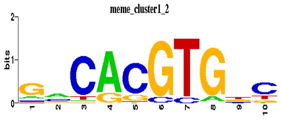

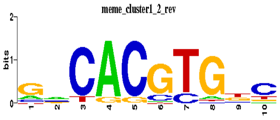

forward *reverse compliment*

Name	*E value*	*Alignment*	*Motif*
ABFs	4.9057e-10	GNCACGTGNC<br>--CACGTGGC	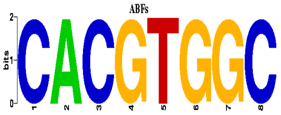
ABRE	8.6656e-09	GNCACGTGNC<br>--YACGTGGC	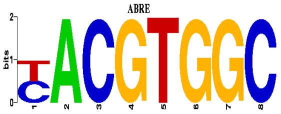
G-box	4.7974e-08	GNCACGTGNC<br>--CACGTG--	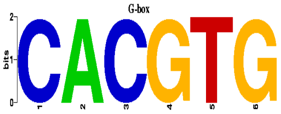
ABRE-like	6.4696e-08	GNCACGTGNC<br>KMCACGTN--	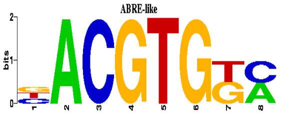

CBF2	7.6291e-08	GNCACGTGNC<br>-CCACGTGG-	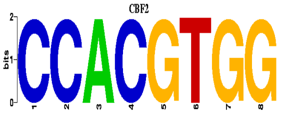

con_10_cluster1_2 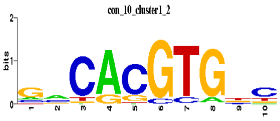 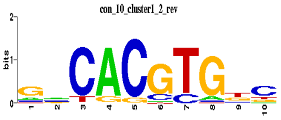

forward *reverse compliment*

Name	*E value*	*Alignment*	*Motif*
ABFs	3.5788e-10	NNCACGTGNC<br>GCCACGTG--	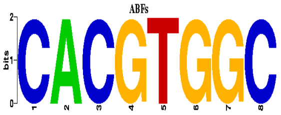
ABRE	8.4367e-09	NNCACGTGNC<br>GCCACGTR--	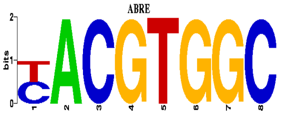
G-box	4.5297e-08	GNCACGTGNN<br>--CACGTG--	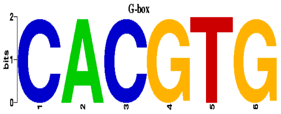
ABRE-like	6.7466e-08	NNCACGTGNC<br>KMCACGTN--	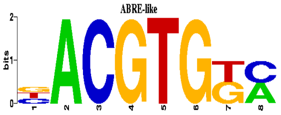
CBF2	1.0407e-07	NNCACGTGNC<br>-CCACGTGG-	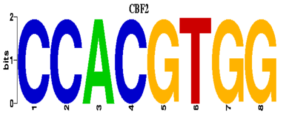

aa_10_cluster1_3 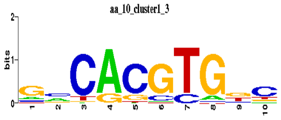 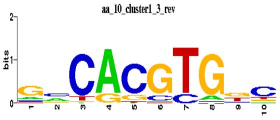

forward *reverse compliment*

Name	*E value*	*Alignment*	*Motif*

ABFs	1.2256e-10	GNCACGTGNC<br>GCCACGTG--	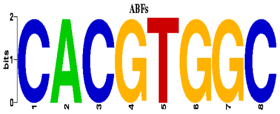
ABRE	2.7637e-09	GNCACGTGNC<br>GCCACGTR--	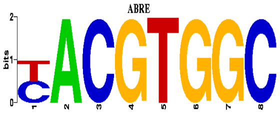
CBF2	5.6029e-09	GNCACGTGNC<br>-CCACGTGG-	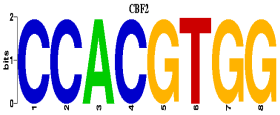
GBF1_2_3	5.6029e-09	GNCACGTGNC<br>-CCACGTGG-	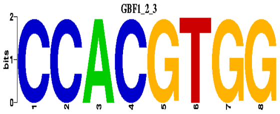
G-box	5.3475e-08	GNCACGTGNC<br>--CACGTG--	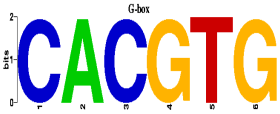

---

meme_cluster2_5

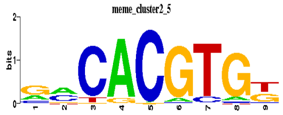

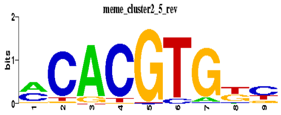

forward *reverse compliment*

Name	*E value*	*Alignment*	*Motif*
ABFs	3.6560e-11	MCACGTGKY<br>-CACGTGGC	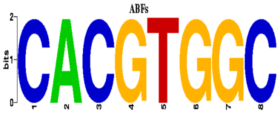
ABRE	1.2097e-09	RMCACGTGK<br>GCCACGTR-	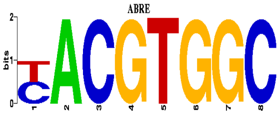
CBF2	2.4678e-09	MCACGTGKY<br>CCACGTGG-	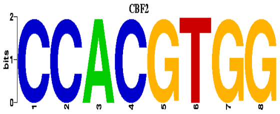
GBF1_2_3	2.4678e-09	MCACGTGKY<br>CCACGTGG-	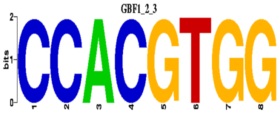
G-box	1.4540e-08	MCACGTGKY<br>-CACGTG--	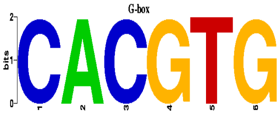

meme_cluster4_2

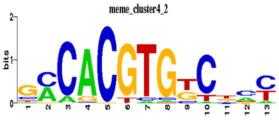 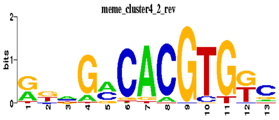

forward *reverse compliment*

Name	*E value*	*Alignment*	*Motif*
ABFs	4.5627e-10	GMCACGTGKCNMY<br>GCCACGTG-----	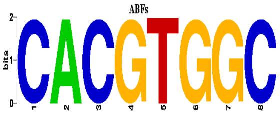
ABRE	1.5589e-08	GMCACGTGKCNMY<br>GCCACGTR-----	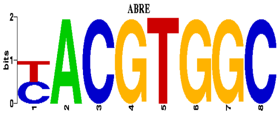
CBF2	6.5834e-08	GMCACGTGKCNMY<br>-CCACGTGG----	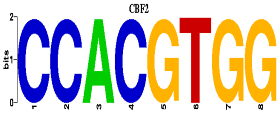
GBF1_2_3	6.5834e-08	GMCACGTGKCNMY<br>-CCACGTGG----	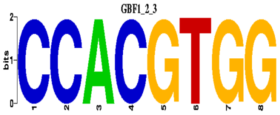
G-box	1.3804e-07	GMCACGTGKCNMY<br>--CACGTG-----	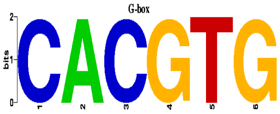

meme_cluster4_4

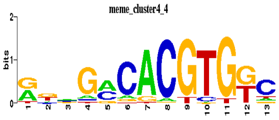 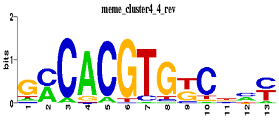

forward *reverse compliment*

Name	*E value*	*Alignment*	*Motif*
ABFs	2.9884e-10	GMCACGTGKCNNY<br>GCCACGTG-----	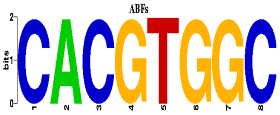
ABRE	1.5176e-08	GMCACGTGKCNNY<br>GCCACGTR-----	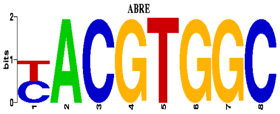

CBF2	2.9740e-08	RNNGMCACGTGKC<br>----CCACGTGG-	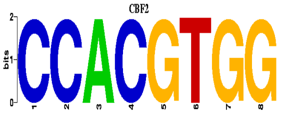
GBF1_2_3	2.9740e-08	RNNGMCACGTGKC<br>----CCACGTGG-	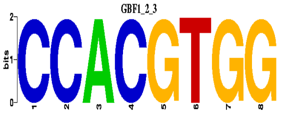
G-box	1.3818e-07	GMCACGTGKCNNY<br>--CACGTG-----	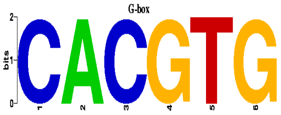

---

meme_cluster4_5

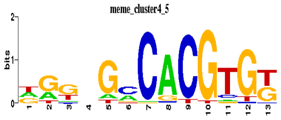

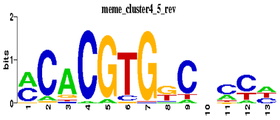

forward *reverse compliment*

Name	*E value*	*Alignment*	*Motif*
ABFs	8.9105e-11	NGKNGMCACGTGK<br>----GCCACGTG-	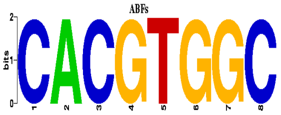
CBF2	4.6778e-09	NGKNGMCACGTGK<br>-----CCACGTGG	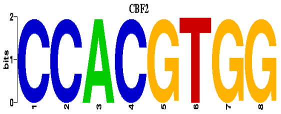
GBF1_2_3	4.6778e-09	NGKNGMCACGTGK<br>-----CCACGTGG	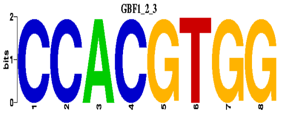
ABRE	6.8482e-09	NGKNGMCACGTGK<br>----GCCACGTR-	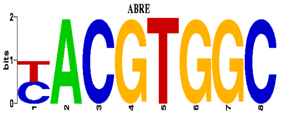
G-box	1.3064e-07	MCACGTGKCNMCN<br>-CACGTG------	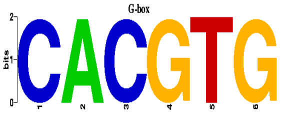

---

con_10_cluster4_2

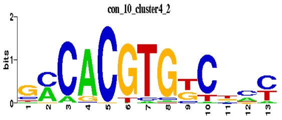

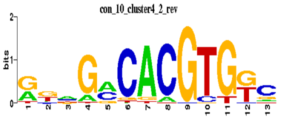

forward *reverse compliment*

Name	*E value*	*Alignment*	*Motif*
ABFs	4.5627e-10	GMCACGTGKCNMY<br>GCCACGTG-----	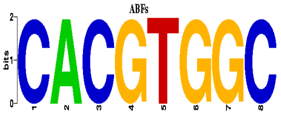
ABRE	1.5589e-08	GMCACGTGKCNMY<br>GCCACGTR-----	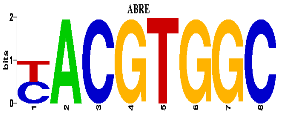
CBF2	6.5834e-08	GMCACGTGKCNMY<br>-CCACGTGG----	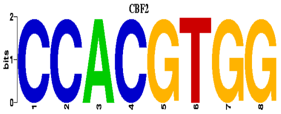
GBF1_2_3	6.5834e-08	GMCACGTGKCNMY<br>-CCACGTGG----	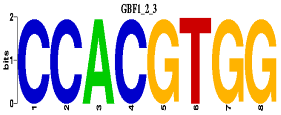
G-box	1.3804e-07	GMCACGTGKCNMY<br>--CACGTG-----	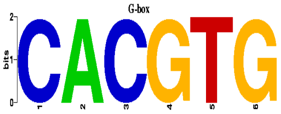

---

aa_10_cluster4_5

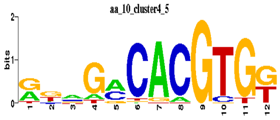

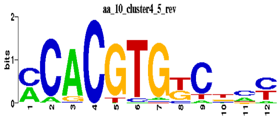

forward *reverse compliment*

Name	*E value*	*Alignment*	*Motif*
ABFs	2.7376e-09	RKNGMCACGTGK<br>---GCCACGTG-	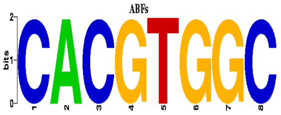
CBF2	2.6624e-08	MCACGTGKCNMY<br>CCACGTGG----	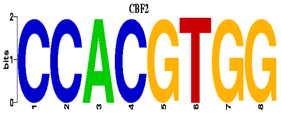
GBF1_2_3	2.6624e-08	MCACGTGKCNMY<br>CCACGTGG----	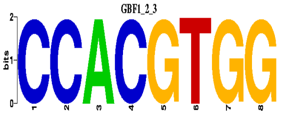
G-box	1.0105e-07	RKNGMCACGTGK<br>-----CACGTG-	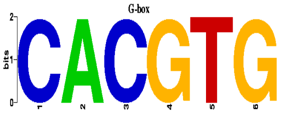

ABRE	1.1506e-07	RKNGMCACGTGK<br>---GCCACGTR-	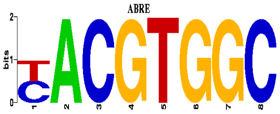

biop_10_cluster4_1 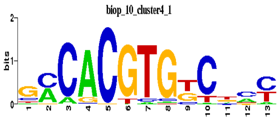

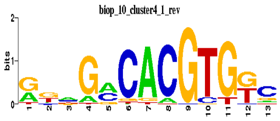

forward *reverse compliment*

Name	*E value*	*Alignment*	*Motif*
ABFs	4.5627e-10	GMCACGTGKCNMY<br>GCCACGTG-----	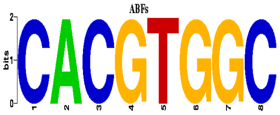
ABRE	1.5589e-08	GMCACGTGKCNMY<br>GCCACGTR-----	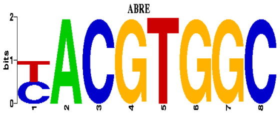
CBF2	6.5834e-08	GMCACGTGKCNMY<br>-CCACGTGG----	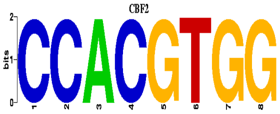
GBF1_2_3	6.5834e-08	GMCACGTGKCNMY<br>-CCACGTGG----	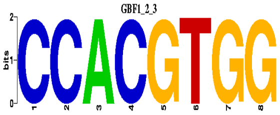
G-box	1.3804e-07	GMCACGTGKCNMY<br>--CACGTG-----	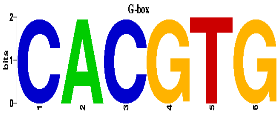

biop_10_cluster4_2 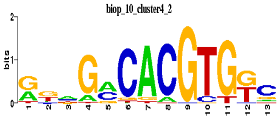

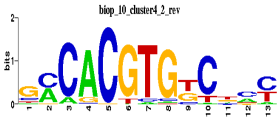

forward *reverse compliment*

Name	*E value*	*Alignment*	*Motif*

ABFs	4.5627e-10	GMCACGTGKCNMY<br>GCCACGTG-----	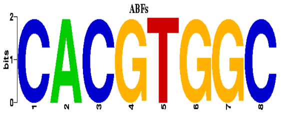
ABRE	1.5589e-08	GMCACGTGKCNMY<br>GCCACGTR-----	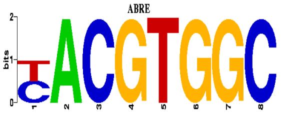
CBF2	6.5834e-08	RKNGMCACGTGKC<br>----CCACGTGG-	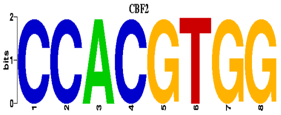
GBF1_2_3	6.5834e-08	RKNGMCACGTGKC<br>----CCACGTGG-	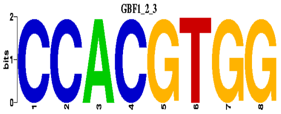
G-box	1.3804e-07	GMCACGTGKCNMY<br>--CACGTG-----	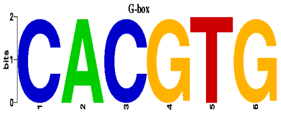

---

biop_10_cluster4_4

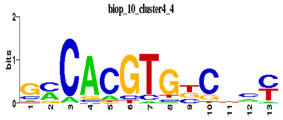

forward

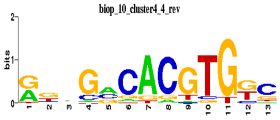

reverse compliment

Name	*E value*	*Alignment*	*Motif*
ABFs	3.9958e-10	GMCACGTGNCNNY<br>GCCACGTG-----	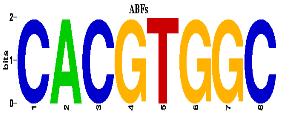
ABRE	2.3938e-08	GMCACGTGNCNNY<br>GCCACGTR-----	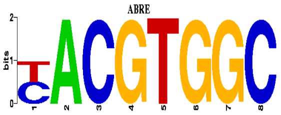
G-box	1.5295e-07	GMCACGTGNCNNY<br>--CACGTG-----	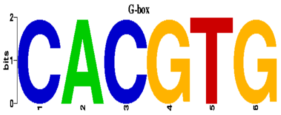
CBF2	1.6829e-07	GMCACGTGNCNNY<br>-CCACGTGG----	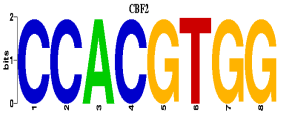

GBF1_2_3	1.6829e-07	GMCACGTGNCNNY<br>-CCACGTGG----	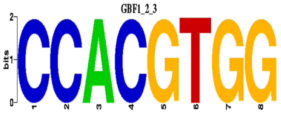

---

aa_10_cluster2_5

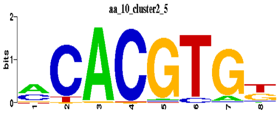

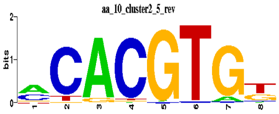

forward *reverse compliment*

Name	*E value*	*Alignment*	*Motif*
G-box	4.2821e-09	MCACGTGK<br>-CACGTG-	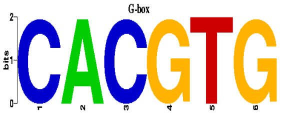
CBF2	5.1085e-09	MCACGTGK<br>CCACGTGG	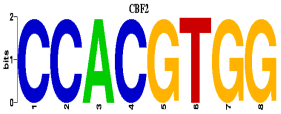
GBF1_2_3	5.1085e-09	MCACGTGK<br>CCACGTGG	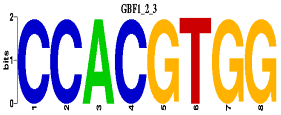
ABFs	5.7683e-08	MCACGTGK-<br>-CACGTGGC	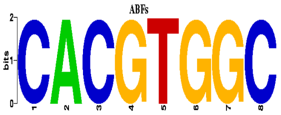
ABRE-like	7.0439e-08	-MCACGTGK<br>KMCACGTN-	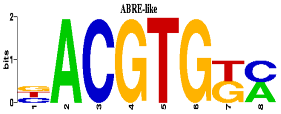

---

meme_cluster2_1

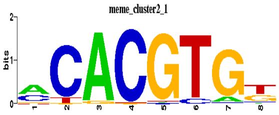

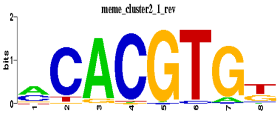

forward *reverse compliment*

Name	*E value*	*Alignment*	*Motif*
G-box	4.3084e-09	MCACGTGK<br>-CACGTG-	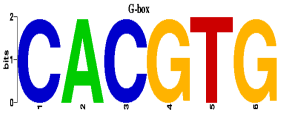

CBF2	8.5899e-09	MCACGTGK<br>CCACGTGG	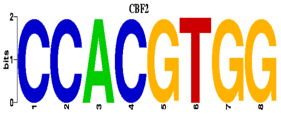
GBF1_2_3	8.5899e-09	MCACGTGK<br>CCACGTGG	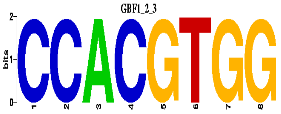
ABRE-like	7.9632e-08	-MCACGTGK<br>KMCACGTN-	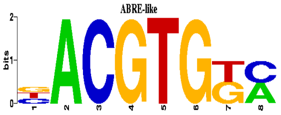
Z-box	9.0409e-08	MCACGTGK<br>ACACGTAT	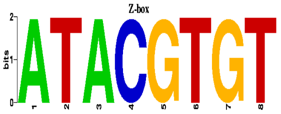

---

biop_10_all_clust_v4

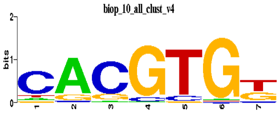

forward

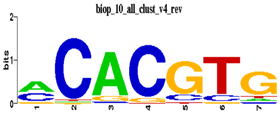

reverse compliment

Name	*E value*	*Alignment*	*Motif*
G-box	1.2324e-09	MCACGTG<br>-CACGTG	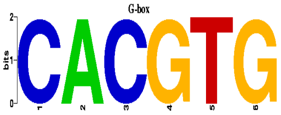
ABFs	6.7752e-09	-MCACGTG<br>GCCACGTG	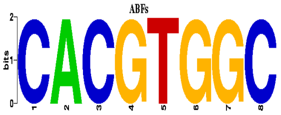
CBF2	6.7752e-09	MCACGTG-<br>CCACGTGG	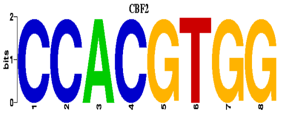
GBF1_2_3	6.7752e-09	MCACGTG-<br>CCACGTGG	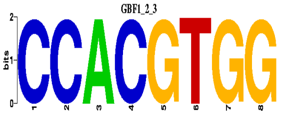
ABRE-like	1.8273e-08	-MCACGTG<br>KMCACGTN	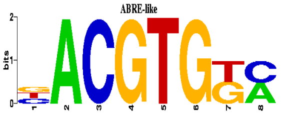

---

meme_all_cluster_1

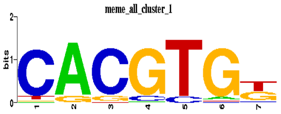

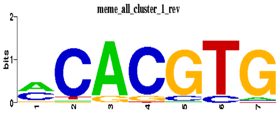

forward *reverse compliment*

Name	*E value*	*Alignment*	*Motif*
G-box	1.1774e-09	CACGTGK<br>CACGTG-	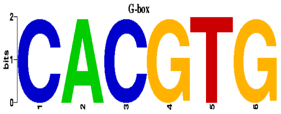
ABFs	2.2400e-09	-MCACGTG<br>GCCACGTG	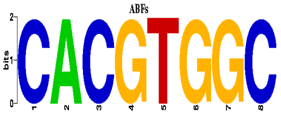
CBF2	2.2400e-09	MCACGTG-<br>CCACGTGG	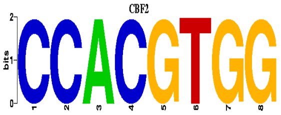
GBF1_2_3	2.2400e-09	MCACGTG-<br>CCACGTGG	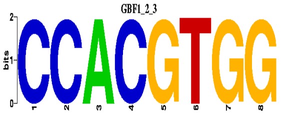
ABRE-like	9.5612e-09	-MCACGTG<br>KMCACGTN	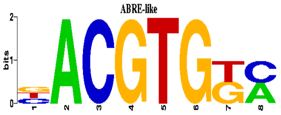

---

con_10_cluster1_5

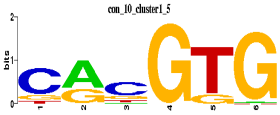

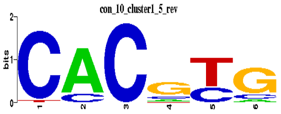

forward *reverse compliment*

Name	*E value*	*Alignment*	*Motif*
G-box	4.5925e-10	CACGTG<br>CACGTG	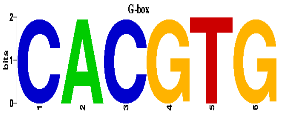
ABFs	2.9924e-08	--CACGTG<br>GCCACGTG	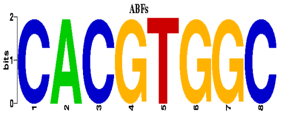

CBF2	2.9924e-08	-CACGTG-<br>CCACGTGG	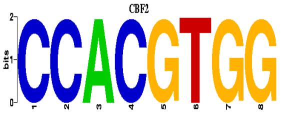
GBF1_2_3	2.9924e-08	-CACGTG-<br>CCACGTGG	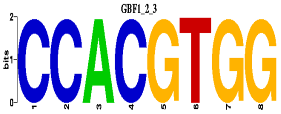
ABRE	9.0519e-07	--CACGTG<br>GCCACGTR	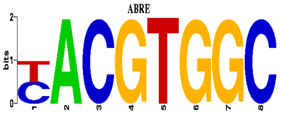

---

biop_10_all_clust_v3

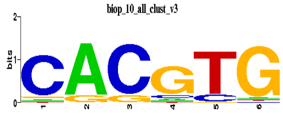

forward

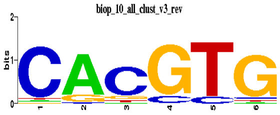

reverse compliment

Name	*E value*	*Alignment*	*Motif*
G-box	6.2438e-11	CACGTG<br>CACGTG	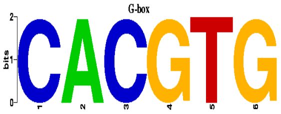
ABFs	5.4690e-09	--CACGTG<br>GCCACGTG	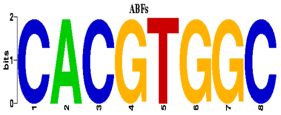
CBF2	5.4690e-09	-CACGTG-<br>CCACGTGG	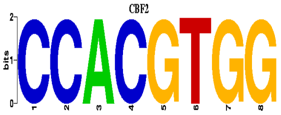
GBF1_2_3	5.4690e-09	-CACGTG-<br>CCACGTGG	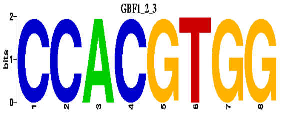
ABRE	2.4512e-07	--CACGTG<br>GCCACGTR	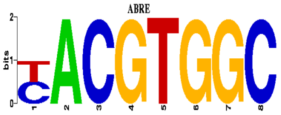

---

meme_all_cluster_4

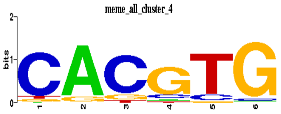

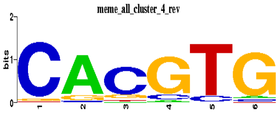

forward *reverse compliment*

Name	*E value*	*Alignment*	*Motif*
G-box	5.4745e-11	CACGTG<br>CACGTG	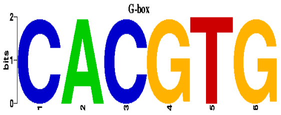
ABFs	4.8887e-09	--CACGTG<br>GCCACGTG	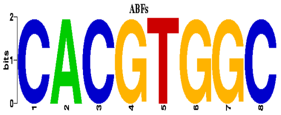
CBF2	4.8887e-09	-CACGTG-<br>CCACGTGG	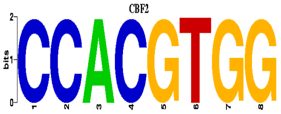
GBF1_2_3	4.8887e-09	-CACGTG-<br>CCACGTGG	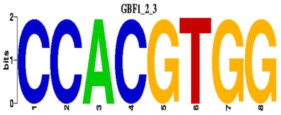
ABRE	1.9855e-07	--CACGTG<br>GCCACGTR	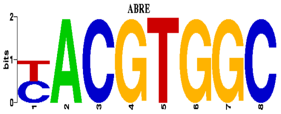

---

meme_all_cluster_3

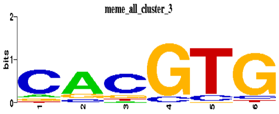

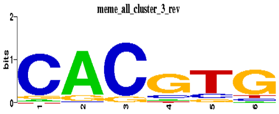

forward *reverse compliment*

Name	*E value*	*Alignment*	*Motif*
G-box	5.9453e-11	CACGTG<br>CACGTG	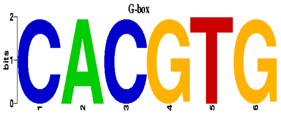
ABFs	5.2451e-09	--CACGTG<br>GCCACGTG	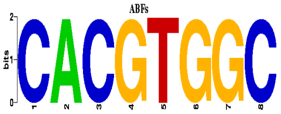
CBF2	5.2451e-09	-CACGTG-<br>CCACGTGG	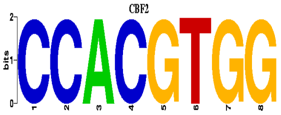
GBF1_2_3	5.2451e-09	-CACGTG-<br>CCACGTGG	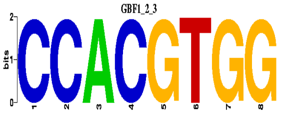

ABRE	3.3526e-07	--CACGTG<br>GCCACGTR	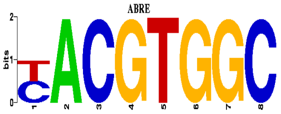

biop_10_cluster2_4

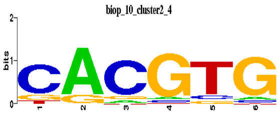

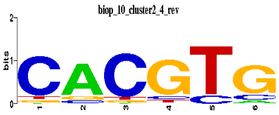

forward *reverse compliment*

Name	*E value*	*Alignment*	*Motif*
G-box	6.9934e-11	CACGTG<br>CACGTG	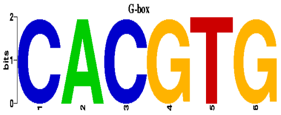
ABFs	6.0243e-09	--CACGTG<br>GCCACGTG	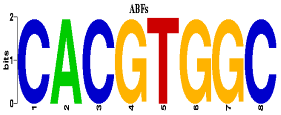
CBF2	6.0243e-09	-CACGTG-<br>CCACGTGG	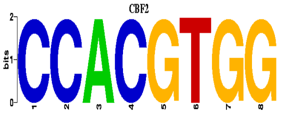
GBF1_2_3	6.0243e-09	-CACGTG-<br>CCACGTGG	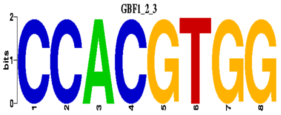
ABRE	2.2064e-07	--CACGTG<br>GCCACGTR	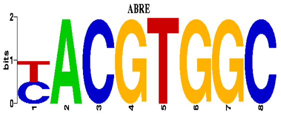

meme_cluster4_1

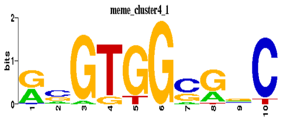

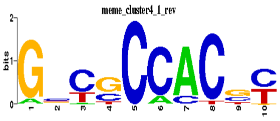

forward *reverse compliment*

Name	*E value*	*Alignment*	*Motif*

ERF1	3.7384e-07	RNGTGGSGNC<br>----GGCGGC	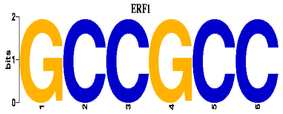
GCC-box	3.7384e-07	RNGTGGSGNC<br>----GGCGGC	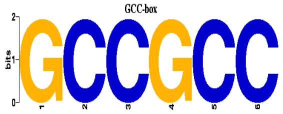
ABFs	7.9180e-06	GNCSCCACNY-<br>---GCCACGTG	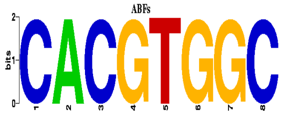
ABRE	7.9180e-06	GNCSCCACNY-<br>---GCCACGTR	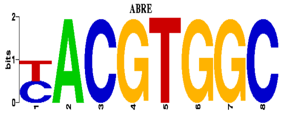
SORLIP1	1.9452e-04	RNGTGGSGNC<br>--GTGGCT--	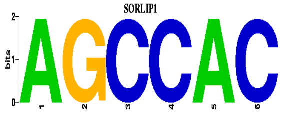

---

con_10_cluster4_1

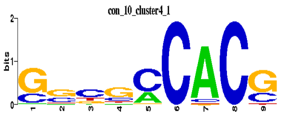

forward

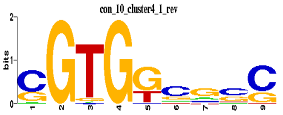

reverse compliment

Name	*E value*	*Alignment*	*Motif*
ABFs	2.5416e-05	GSNSCCACG--<br>---GCCACGTG	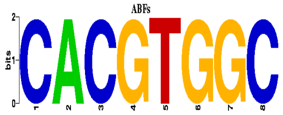
ABRE	2.5416e-05	GSNSCCACG--<br>---GCCACGTR	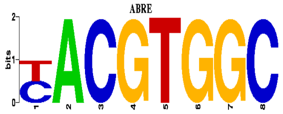
ERF1	5.4767e-05	CGTGGSNSC<br>---GGCGGC	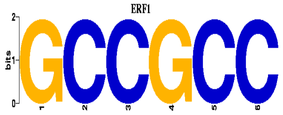
GCC-box	5.4767e-05	CGTGGSNSC<br>---GGCGGC	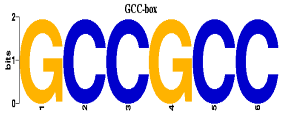

SORLIP1	1.0639e-04	CGTGGSNSC<br>-GTGGCT--	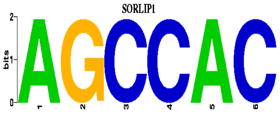

---

con_10_cluster4_4 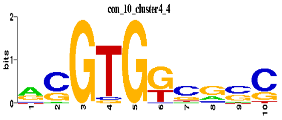

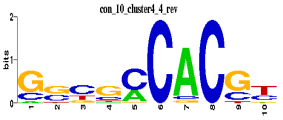

forward *reverse compliment*

Name	*E value*	*Alignment*	*Motif*
ABFs	1.2568e-07	GSYGCCACGY-<br>---GCCACGTG	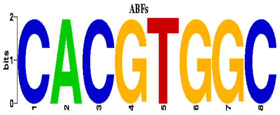
ABRE	1.2568e-07	GSYGCCACGY-<br>---GCCACGTR	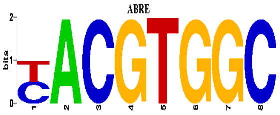
ABRE-like	2.9189e-06	GSYGCCACGY-<br>---KMCACGTN	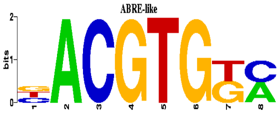
ERF1	5.3387e-05	RCGTGGCRSC<br>----GGCGGC	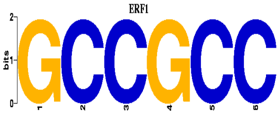
GCC-box	5.3387e-05	RCGTGGCRSC<br>----GGCGGC	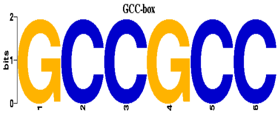

---

biop_10_cluster4_3 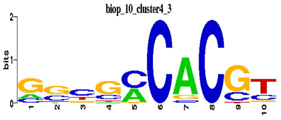

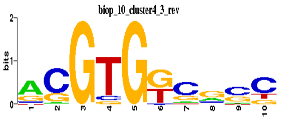

forward *reverse compliment*

Name	*E value*	*Alignment*	*Motif*

ABFs	2.0624e-07	GSNGMCACGT-<br>---GCCACGTG	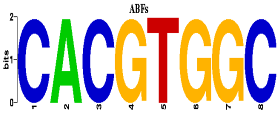
ABRE	2.0624e-07	GSNGMCACGT-<br>---GCCACGTR	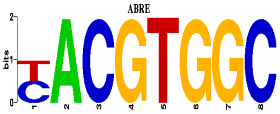
ABRE-like	1.2093e-06	GSNGMCACGT-<br>---KMCACGTN	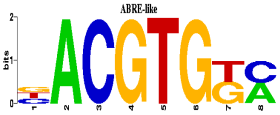
ACE	6.6804e-05	GSNGMCACGT---<br>---GACACGTAGA	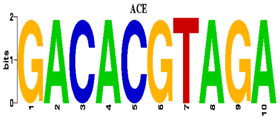
CBF2	1.2243e-04	GSNGMCACGT--<br>----CCACGTGG	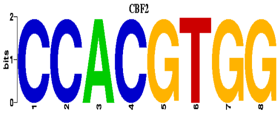

---

con_10_cluster4_5

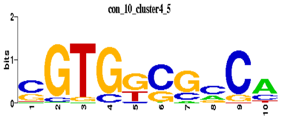

forward

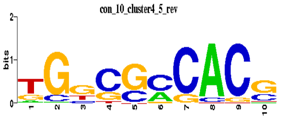

reverse compliment

Name	*E value*	*Alignment*	*Motif*
ABFs	1.3922e-04	TGKCGMCACS--<br>----GCCACGTG	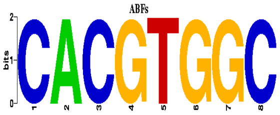
ABRE	1.3922e-04	TGKCGMCACS--<br>----GCCACGTR	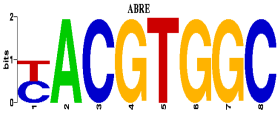
SORLIP1	4.0606e-04	SGTGKCGMCA<br>-GTGGCT---	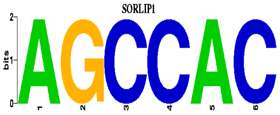
ABRE-like	1.5073e-03	TGKCGMCACS--<br>----KMCACGTN	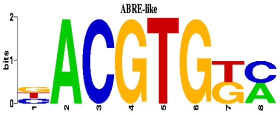

ERF1	2.4920e-03	TGKCGMCACS<br>-GCCGCC---	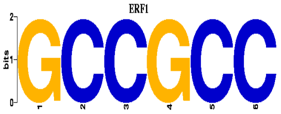

con_10_cluster4_3

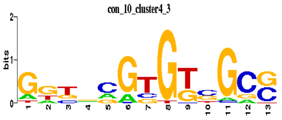

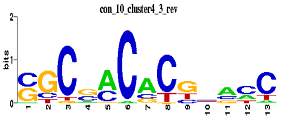

forward *reverse compliment*

Name	*E value*	*Alignment*	*Motif*
ACE	6.2490e-05	SGCSACACKNNNC<br>---GACACGTAGA	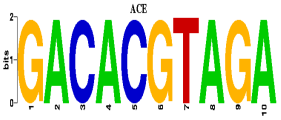
Z-box	1.1623e-04	SGCSACACKNNNC<br>----ACACGTAT-	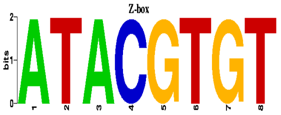
ABRE-like	1.2366e-04	SGCSACACKNNNC<br>---KMCACGTN--	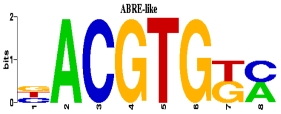
ABRE	1.7706e-04	SGCSACACKNNNC<br>---GCCACGTR--	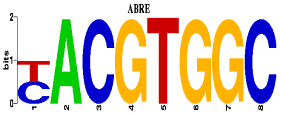
ABFs	4.4369e-03	SGCSACACKNNNC<br>---GCCACGTG--	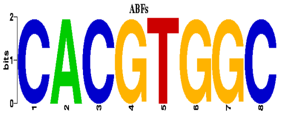

biop_10_cluster4_5

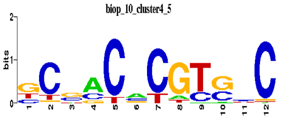

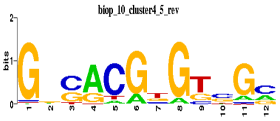

forward *reverse compliment*

Name	*E value*	*Alignment*	*Motif*

ABFs	2.9123e-06	GNSACGNGKNGN<br>GCCACGTG----	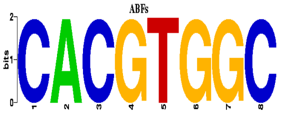
G-box	3.4761e-06	GNSACGNGKNGN<br>--CACGTG----	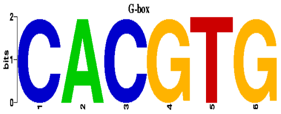
ABRE-like	3.8698e-06	NCNMCNCGTSNC<br>--KMCACGTN--	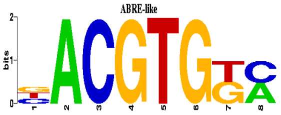
ABRE	2.3570e-05	GNSACGNGKNGN<br>GCCACGTR----	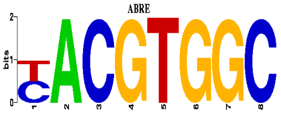
ACE	1.1705e-04	GNSACGNGKNGN<br>TCTACGTGTC--	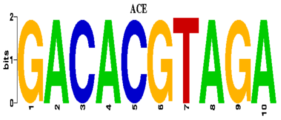

---

biop_10_cluster2_3

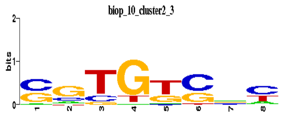

forward

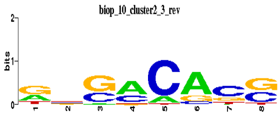

reverse compliment

Name	*E value*	*Alignment*	*Motif*
ACE	6.1809e-04	----SGTGKSNY<br>TCTACGTGTC--	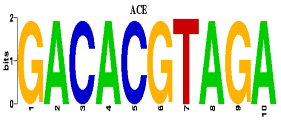
ABRE-like	2.8663e-03	RNSMCACS--<br>--KMCACGTN	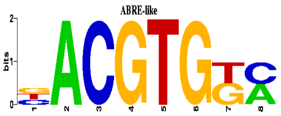
ABFs	4.1135e-03	RNSMCACS--<br>--GCCACGTG	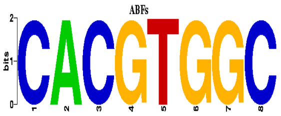
ABRE	4.1135e-03	RNSMCACS--<br>--GCCACGTR	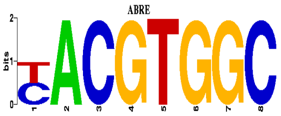

Z-box	6.8578e-03	RNSMCACS---<br>---ACACGTAT	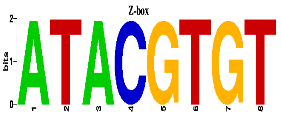

meme_cluster2_3

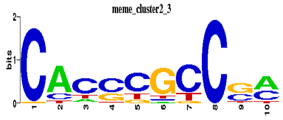

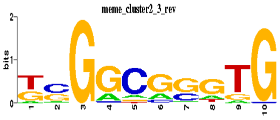

forward *reverse compliment*

Name	*E value*	*Alignment*	*Motif*
E2F-varient	3.9617e-06	KSGGCGSGTG-<br>--GGCGGGAGA	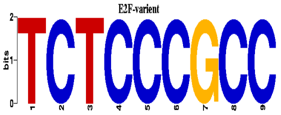
ERF1	2.4954e-04	KSGGCGSGTG<br>--GGCGGC--	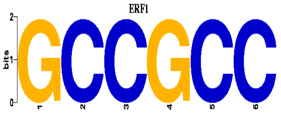
GCC-box	2.4954e-04	KSGGCGSGTG<br>--GGCGGC--	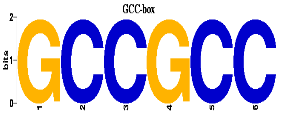
RAV1-B	3.7257e-04	CACSCGCCSM<br>CACCTG----	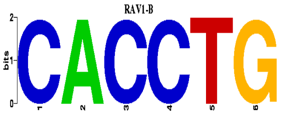
Hexamer	1.0673e-03	CACSCGCCSM<br>---CCGTCG-	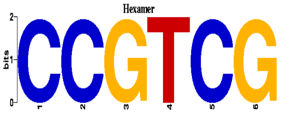

meme_cluster2_2

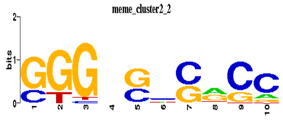

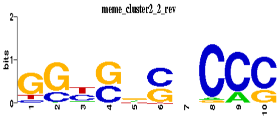

forward *reverse compliment*

Name	*E value*	*Alignment*	*Motif*
ERF1	1.3269e-04	GGYSNSNCCC<br>GGCGGC----	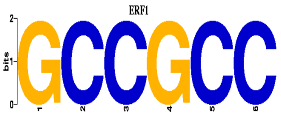

GCC-box	1.3269e-04	GGYSNSNCCC<br>GGCGGC----	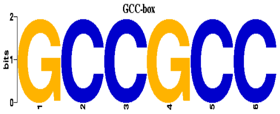
SORLIP2	1.1345e-03	GGYSNSNCCC<br>---GGCCC--	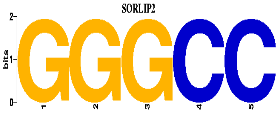
CBF1	1.8444e-03	GGYSNSNCCC<br>-GTCGGCCA-	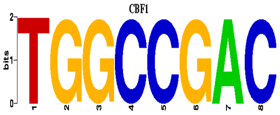
ERE	7.7552e-03	GGYSNSNCCC-<br>GGCGGCTCTTA	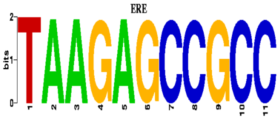

biop_10_cluster3_1

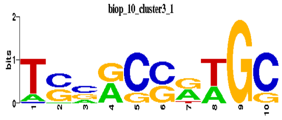

forward

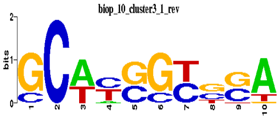

reverse compliment

Name	*E value*	*Alignment*	*Motif*
CBF1	8.9174e-03	-TSNRCSRTGC<br>GTCGGCCA---	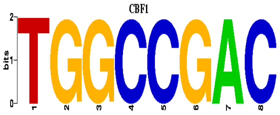
octamer	3.9962e-02	--TSNRCSRTGC<br>GATCCGCG----	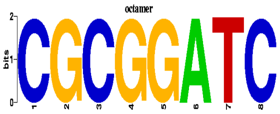
ERF1	7.1048e-02	GCAYSGYNSA<br>----GGCGGC	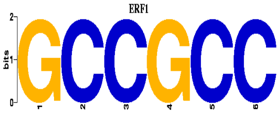
GCC-box	7.1048e-02	GCAYSGYNSA<br>----GGCGGC	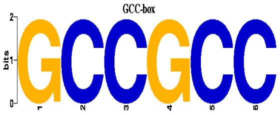
RY-repeat	8.6815e-02	---GCAYSGYNSA<br>CATGCATG-----	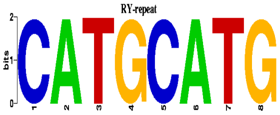

con_10_cluster3_1

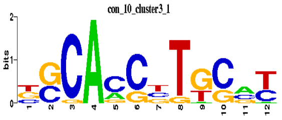

forward

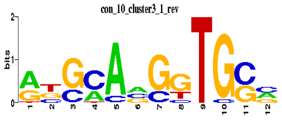

reverse compliment

Name	*E value*	*Alignment*	*Motif*
CArG2	1.7770e-03	NSCACCNTGCNY------<br>CTTACCTTTCATGGATTA	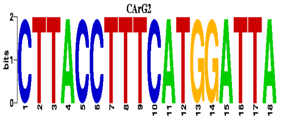
RAV1-B	5.3561e-03	RNGCANGGTGSN<br>----CAGGTG--	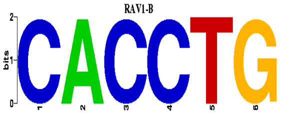
ABFs	6.6080e-03	NSCACCNTGCNY<br>--CACGTGGC--	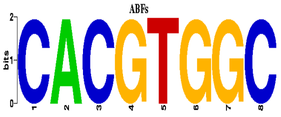
EIN3	7.5321e-03	---------------NSCACCNTGCNY--<br>GGATTCAAGATACATGCCCCCTTGAATCC	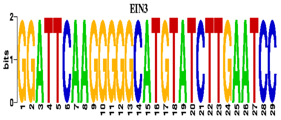
ABRE	3.2934e-02	RNGCANGGTGSN<br>--GCCACGTR--	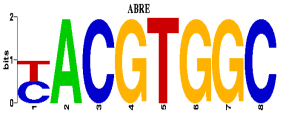

---

con_10_cluster3_2

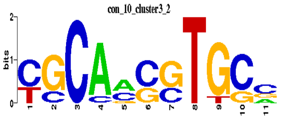

forward

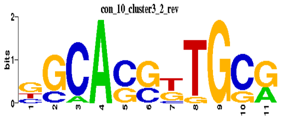

reverse compliment

Name	*E value*	*Alignment*	*Motif*
ABFs	1.3651e-05	NGCACGKTGCG<br>--CACGTGGC-	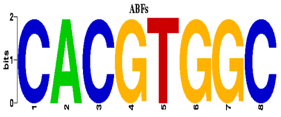
ABRE	2.2141e-04	CGCAMCGTGCN<br>-GCCACGTR--	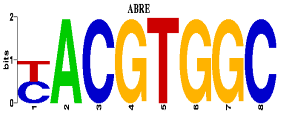

CBF2	2.2302e-03	CGCAMCGTGCN<br>--CCACGTGG-	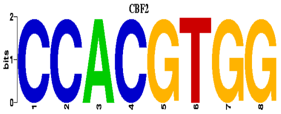
GBF1_2_3	2.2302e-03	CGCAMCGTGCN<br>--CCACGTGG-	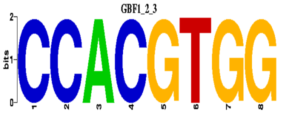
G-box	4.1144e-03	NGCACGKTGCG<br>--CACGTG---	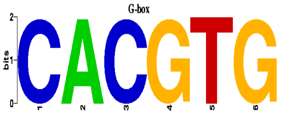

aa_10_cluster3_1

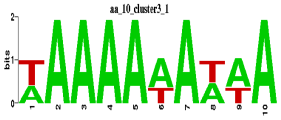

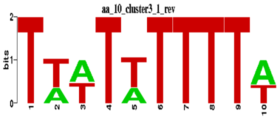

forward *reverse compliment*

Name	*E value*	*Alignment*	*Motif*
CCA1_v3	6.6562e-06	-------TTWTTTTTTW<br>TCATAGATTTTTTTT--	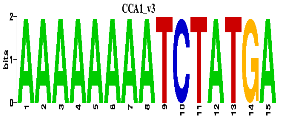
EveningElement	1.8741e-04	TTWTTTTTTW<br>AGATATTTT-	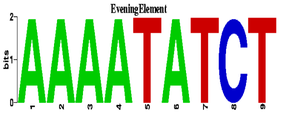
AP1	1.3961e-03	-WAAAAAAWAA<br>CTAAAAATGG-	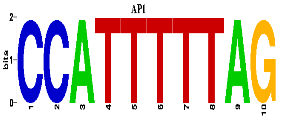
CCA1	2.2145e-03	TTWTTTTTTW<br>AGATTKTT--	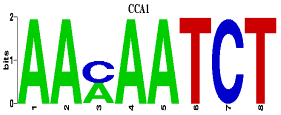
AG_v3	2.4127e-03	WAAAAAAWAA<br>CCAAAAATGG	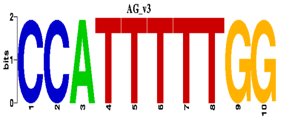

aa_10_all_cluster_1

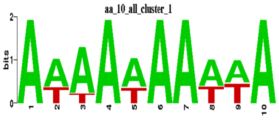

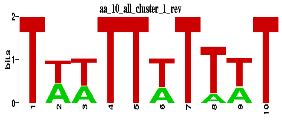

forward *reverse compliment*

Name	*E value*	*Alignment*	*Motif*
CCA1_v3	7.1473e-08	-----TWTTTTTTTT<br>TCATAGATTTTTTTT	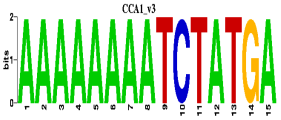
AG_v4	1.1289e-03	-----TWTTTTTTTT-<br>TTTCCTATTCTGTTTT	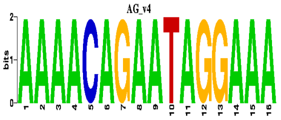
AGL2	2.0531e-03	-TWTTTTTTTT-------<br>NTWWCYAWWWWTGGNWNN	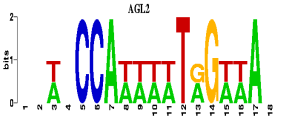
ATHB6	2.6062e-03	AAAAAAAAWA<br>-TAATAATTG	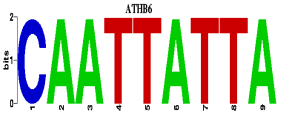
AP1	3.6902e-03	TWTTTTTTTT<br>CCATTTTTAG	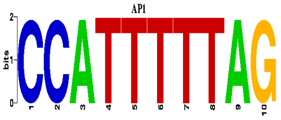

---

aa_10_cluster1_1

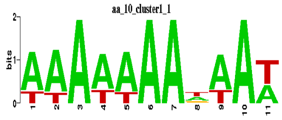

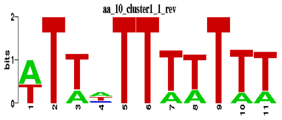

forward *reverse compliment*

Name	*E value*	*Alignment*	*Motif*
CCA1_v3	9.5610e-07	------WTTNTTTTTTT<br>TCATAGATTTTTTTT--	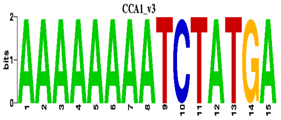
AG_v4	3.8802e-04	------WTTNTTTTTTT<br>TTTCCTATTCTGTTTT-	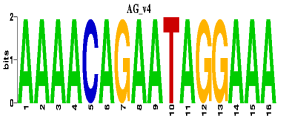
EveningElement	1.6909e-03	AAAAAAANAAW-<br>---AAAATATCT	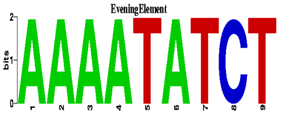
AG_v3	3.1625e-03	AAAAAAANAAW<br>CCAAAAATGG-	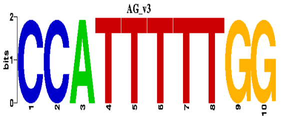

AP1	3.1625e-03	AAAAAAANAAW<br>CTAAAAATGG-	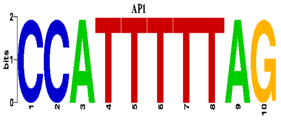

aa_10_cluster2_1

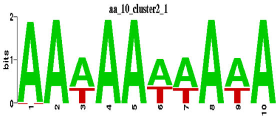

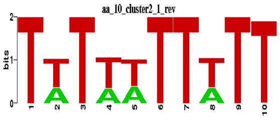

forward *reverse compliment*

Name	*E value*	*Alignment*	*Motif*
CCA1_v3	2.7265e-07	AAAAAAAAAA-----<br>AAAAAAAATCTATGA	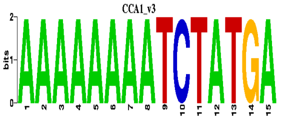
AG_v2	1.4178e-03	AAAAAAAAAA-<br>ACTAAAAATGG	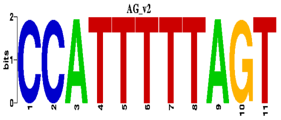
AG_v4	2.6509e-03	-AAAAAAAAAA-----<br>AAAACAGAATAGGAAA	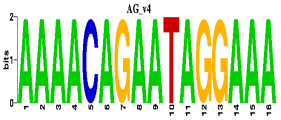
CArG3	3.1380e-03	------AAAAAAAAAA--<br>GTTACTAAAAATGGAAAG	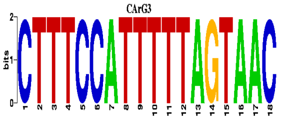
CCA1_v2	3.3213e-03	AAAAAAAAAA<br>AAACAATCTA	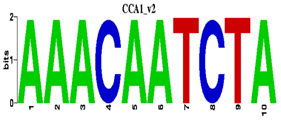

meme_all_cluster_5

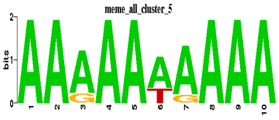

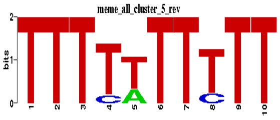

forward *reverse compliment*

Name	*E value*	*Alignment*	*Motif*
CCA1_v3	1.3989e-07	-------TTTTTTTTTT<br>TCATAGATTTTTTTT--	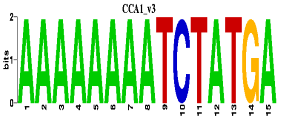

CArG3	4.2201e-04	`------AAAAAAAAAA--`<br>`GTTACTAAAAATGGAAAG`	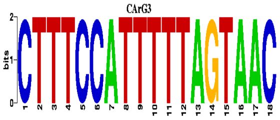
AG_v4	7.4634e-04	`AAAAAAAAAA------`<br>`AAAACAGAATAGGAAA`	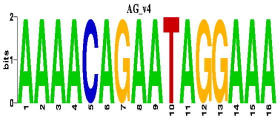
SORLREP2	4.6486e-03	`---TTTTTTTTTT`<br>`ACGTTTTAT----`	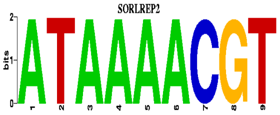
EveningElement	5.2448e-03	`TTTTTTTTTT`<br>`AGATATTTT-`	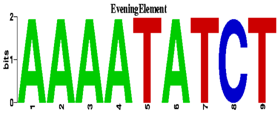

---

aa_10_all_cluster_2

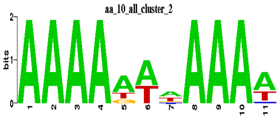

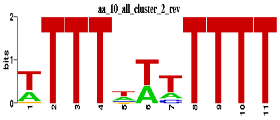

forward *reverse compliment*

Name	*E value*	*Alignment*	*Motif*
CCA1_v3	3.6212e-06	`AAAAAWNAAAW------`<br>`--AAAAAAAATCTATGA`	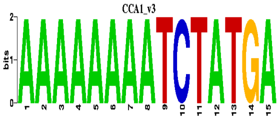
CArG3	5.6222e-05	`------AAAAAWNAAAW-`<br>`GTTACTAAAAATGGAAAG`	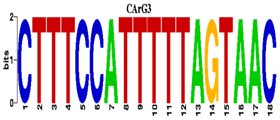
Bellringer_replumless_pennywise	5.2955e-04	`WTTTNWTTTTT`<br>`--YTAATTT--`	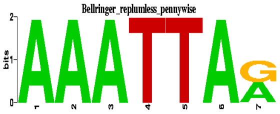
AGL3	3.9670e-03	`-----AAAAAWNAAAW`<br>`TTWCYAWWWWTRGWAA`	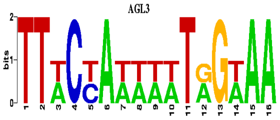
AG_v4	4.7760e-03	`AAAAAWNAAAW-----`<br>`AAAACAGAATAGGAAA`	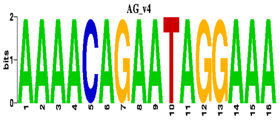

---

aa_10_cluster1_2

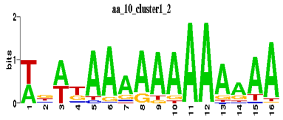

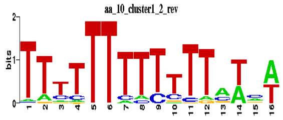

forward *reverse compliment*

Name	*E value*	*Alignment*	*Motif*
CCA1_v3	1.8433e-04	WNWKAAAAAAAAAAAA-------<br>--------AAAAAAAATCTATGA	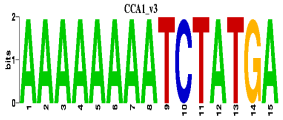
CArG3	3.5288e-04	-WNWKAAAAAAAAAAAA-<br>GTTACTAAAAATGGAAAG	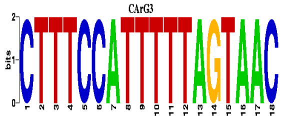
GATA	4.1154e-04	TTTTTTTTTTTTMWNW<br>----------YTATCW	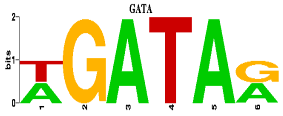
CArG1	6.6231e-04	TTTTTTTTTTTTMWNW--<br>TTTTCCATTTATGTAAAC	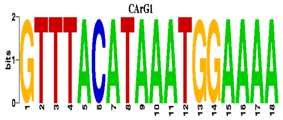
PII	2.0518e-03	-------WNWKAAAAAAAAAAAA<br>TTGGTTTTGATCAAAACCAA---	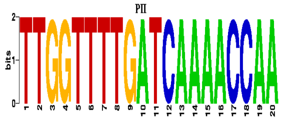

aa_10_cluster3_2

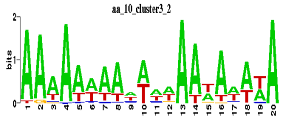

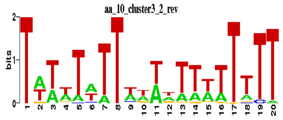

forward *reverse compliment*

Name	*E value*	*Alignment*	*Motif*
CCA1_v3	6.8505e-06	TWTWTWTTWWWNTTWTTTTT<br>----TCATAGATTTTTTTT-	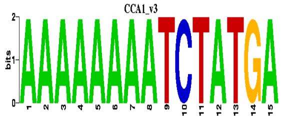
AG_v4	9.2530e-04	AAAAAWAANWWWAAWAWAWA<br>AAAACAGAATAGGAAA----	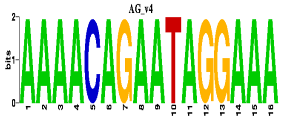

AGL3	2.3682e-03	AAAAAWAANWWWAAWAWAWA<br>-TTWCYAWWWWTRGWAA---	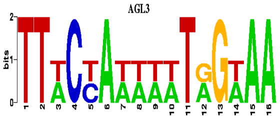
SORLREP3	1.0382e-02	AAAAAWAANWWWAAWAWAWA--<br>-------------ATATATACA	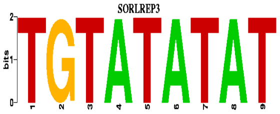
EveningElement	1.0615e-02	TWTWTWTTWWWNTTWTTTTT<br>-AGATATTTT----------	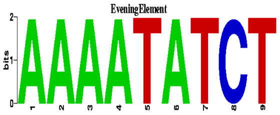

---

aa_10_cluster2_3

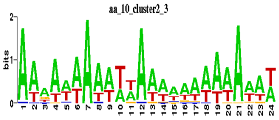

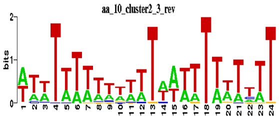

forward *reverse compliment*

Name	*E value*	*Alignment*	*Motif*
CCA1_v3	2.1018e-05	WTWTTTTTWWWTTWATTTTWTNTT<br>----------TCATAGATTTTTTTT	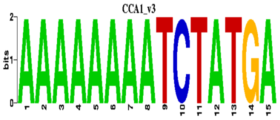
AG_v4	1.0291e-04	AANAWAAAATWAAWWWAAAAAWAW<br>AAAACAGAATAGGAAA--------	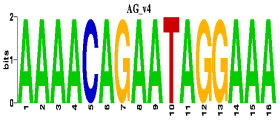
Bellringer_replumless_pennywise	3.1360e-04	WTWTTTTTWWWTTWATTTTWTNTT<br>------------YTAATTT------	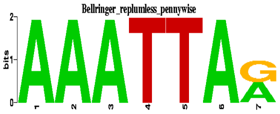
AGL3	2.7876e-03	WTWTTTTTWWWTTWATTTTWTNTT<br>TTWCYAWWWWTRGWAA--------	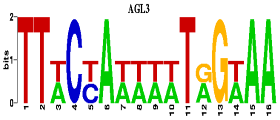
CArG1	6.0236e-03	WTWTTTTTWWWTTWATTTTWTNTT<br>----TTTTCCATTTATGTAAAC--	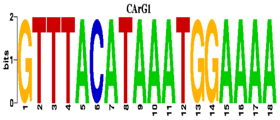

---

aa_10_cluster4_1

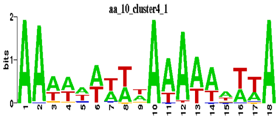

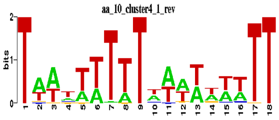

forward *reverse compliment*

Name	*E value*	*Alignment*	*Motif*
Bellringer_replumless_pennywise	9.2666e-04	-TWWNTTTTTWWWWWWWTT<br>YTAATTT------------	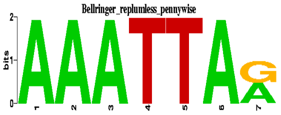
AGL3	1.0540e-03	---AAWWWWWWWWAAAAANWWA<br>TTWCYAWWWWTRGWAA-----	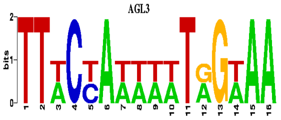
AG	1.4479e-03	-AAWWWWWWWWAAAAANWWA<br>NCCWWWWWWGGNAA-----	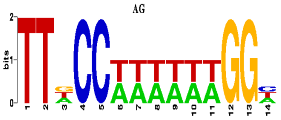
CCA1_v3	2.0689e-03	---TWWNTTTTTWWWWWWWTT<br>TCATAGATTTTTTTT------	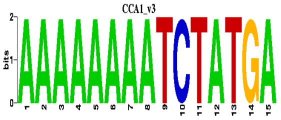
ATHB6	3.8887e-03	TWWNTTTTTWWWWWWWTT<br>TAATAATTG---------	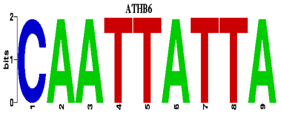

---

aa_10_cluster4_2

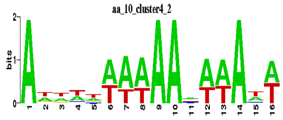

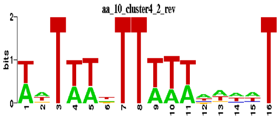

forward *reverse compliment*

Name	*E value*	*Alignment*	*Motif*
CCA1_v3	2.3128e-04	ANNWNWAAAANAWANW-----<br>------AAAAAAAATCTATGA	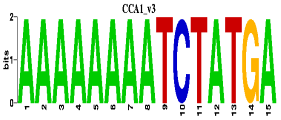
AG	1.5480e-03	-ANNWNWAAAANAWANW<br>NCCWWWWWWGGNAA---	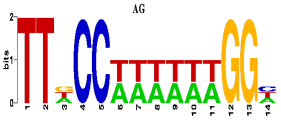
AGL3	1.7961e-03	-----ANNWNWAAAANAWANW<br>TTWCYAWWWWTRGWAA-----	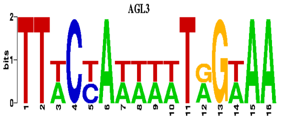
EveningElement	3.7834e-03	ANNWNWAAAANAWANW-<br>--------AAAATATCT	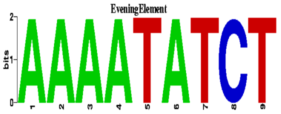

AGL2	7.4264e-03	----ANNWNWAAAANAWANW<br>NNWNCCAWWWWTRGWWAN--	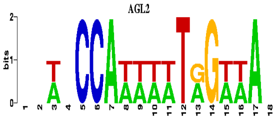

aa_10_cluster4_4

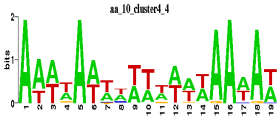

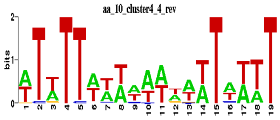

forward *reverse compliment*

Name	*E value*	*Alignment*	*Motif*
AGL3	3.9512e-05	AAWWAWWWWWWWWWWAAWAW<br>---TTWCYAWWWWTRGWAA	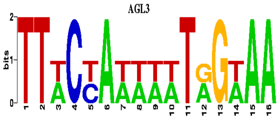
CCA1_v3	7.7605e-04	WTWTTWWWWWWWWWWTWWTT<br>----TCATAGATTTTTTTT	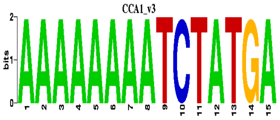
AG	1.3598e-03	WTWTTWWWWWWWWWWTWWTT<br>TTNCCWWWWWWGGN-----	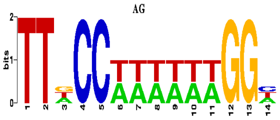
AGL2	1.6321e-03	WTWTTWWWWWWWWWWTWWTT--<br>---NTWWCYAWWWWTGGNWNN	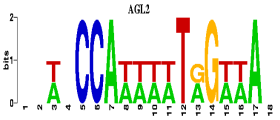
ATHB6	3.0308e-03	WTWTTWWWWWWWWWWTWWTT-<br>-----------TAATAATTG	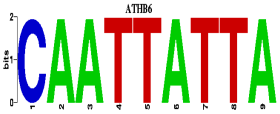

aa_10_cluster2_2

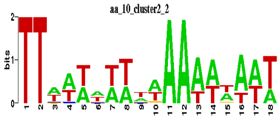

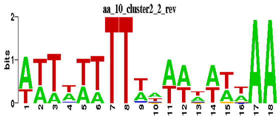

forward *reverse compliment*

Name	*E value*	*Alignment*	*Motif*
AGL3	2.9105e-06	TTWWWWWWNWAAAWWAWT<br>TTWCYAWWWWTRGWAA--	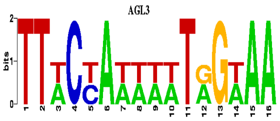

CCA1_v3	1.2740e-03	------AWTWWTTTWNWWWWWWAA<br>TCATAGATTTTTTTT---------	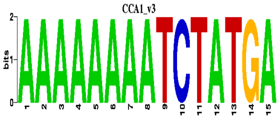
AG	2.9121e-03	-TTWWWWWWNWAAAWWAWT<br>NCCWWWWWWGGNAA-----	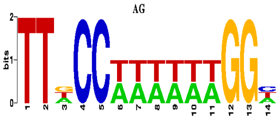
ATHB6	4.2320e-03	AWTWWTTTWNWWWWWWAA<br>---------CAATTATTA-	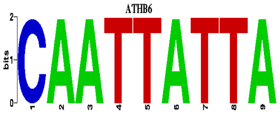
Bellringer_replumless_pennywise	4.4327e-03	TTWWWWWWNWAAAWWAWT<br>YTAATTT-----------	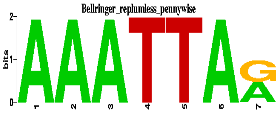

---

aa_10_all_cluster_5

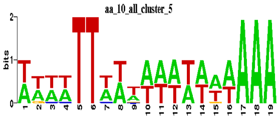

forward

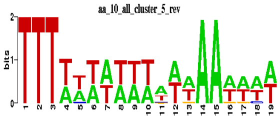

reverse compliment

Name	*E value*	*Alignment*	*Motif*
AGL3	1.6908e-06	WWWWTTWWWWWWWWWAAAA<br>--TTWCYAWWWWTRGWAA-	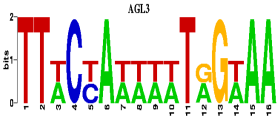
CArG1	4.8204e-04	WWWWTTWWWWWWWWWAAAA<br>-GTTTACATAAATGGAAAA	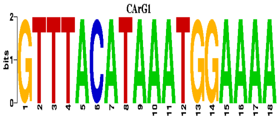
AG	1.7249e-03	WWWWTTWWWWWWWWWAAAA<br>----NCCWWWWWWGGNAA-	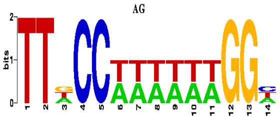
ATHB6	4.9400e-03	WWWWTTWWWWWWWWWAAAA<br>-CAATTATTA---------	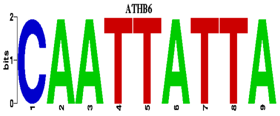
AGL2	7.4895e-03	TTTTWWWWWWWWWAAWWWW<br>-NNWNCCAWWWWTRGWWAN	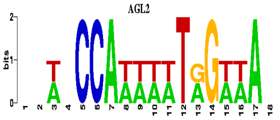

---

aa_10_all_cluster_3

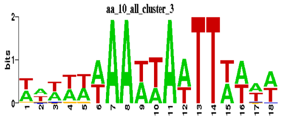

forward

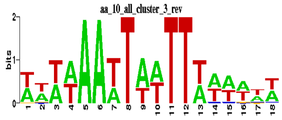

reverse compliment

Name	*E value*	*Alignment*	*Motif*
CCA1_v3	8.7808e-05	-WWWWAATTAWTTWWWWWW<br>TCATAGATTTTTTTT----	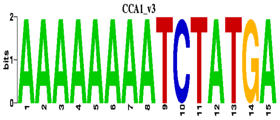
ATHB6	4.9783e-04	WWWWWWAAWTAATTWWWW<br>------TAATAATTG---	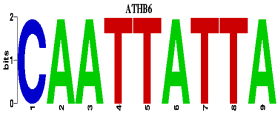
Bellringer_replumless_pennywise	6.8918e-04	WWWWAATTAWTTWWWWWW<br>------YTAATTT-----	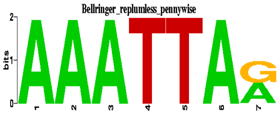
EveningElement	1.3726e-03	WWWWAATTAWTTWWWWWW<br>----AGATATTTT-----	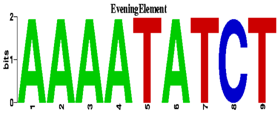
AGL2	1.6064e-03	WWWWAATTAWTTWWWWWW------<br>------NTWWCYAWWWWWTGGNWNN	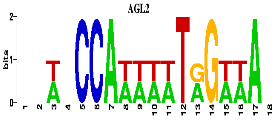

---

aa_10_all_cluster_4

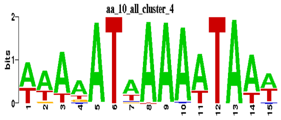

forward

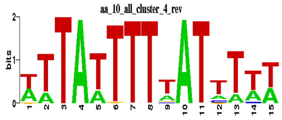

reverse compliment

Name	*E value*	*Alignment*	*Motif*
AG_v2	8.1216e-06	AAAAATAAAAATAAW<br>---ACTAAAAATGG-	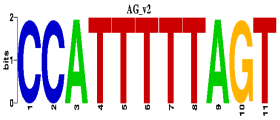
AP1	5.1977e-05	WTTATTTTTATTTTT<br>-CCATTTTTAG----	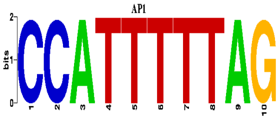

Bellringer_replumless_pennywise	1.9053e-04	WTTATTTTTATTTTT<br>-------YTAATTT--	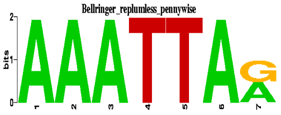
CCA1_v3	2.0639e-04	---WTTATTTTTATTTTT<br>TCATAGATTTTTTTT---	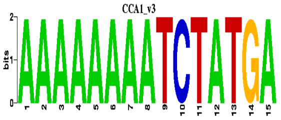
CArG3	1.1132e-03	WTTATTTTTATTTTT------<br>---CTTTCCATTTTTAGTAAC	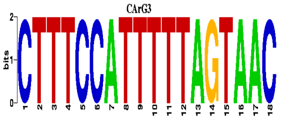

aa_10_cluster2_4

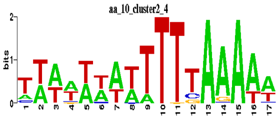

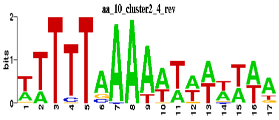

forward *reverse compliment*

Name	*E value*	*Alignment*	*Motif*
AGL3	4.3648e-06	TWWWWWAWTTTTAAAAW<br>-TTWCYAWWWWTRGWAA	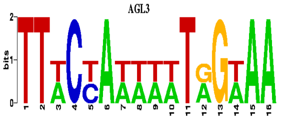
CArG1	2.1833e-04	--TWWWWWAWTTTTAAAAW<br>GTTTACATAAATGGAAAA-	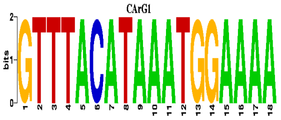
CArG3	2.8140e-04	-WTTTTAAAAWTWWWWWA<br>GTTACTAAAAATGGAAAG	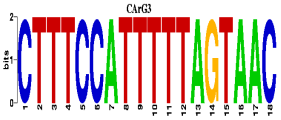
AP1	2.9440e-04	TWWWWWAWTTTTAAAAW<br>----CCATTTTTAG---	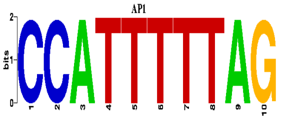
CCA1_v3	8.4278e-04	---TWWWWWAWTTTTAAAAW<br>TCATAGATTTTTTTT-----	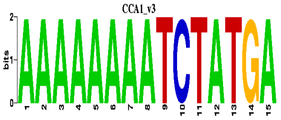

aa_10_cluster4_3

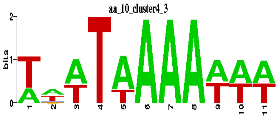

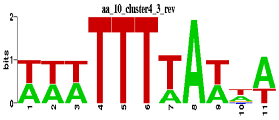

forward *reverse compliment*

Name	*E value*	*Alignment*	*Motif*
CArG3	3.1400e-04	`--TWWTAAAAWWW-----`<br>`GTTACTAAAAATGGAAAG`	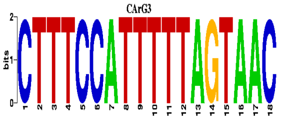
AP1	4.9701e-04	`TWWTAAAAWWW-`<br>`--CTAAAAATGG`	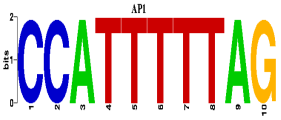
AGL2	7.3177e-04	`-TWWTAAAAWWW------`<br>`NTWWCYAWWWWTGGNWNN`	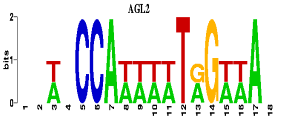
AG_v2	7.5309e-04	`TWWTAAAAWWW-`<br>`-ACTAAAAATGG`	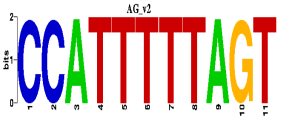
SORLREP2	9.0117e-04	`WWWTTTTAWWA`<br>`ACGTTTTAT--`	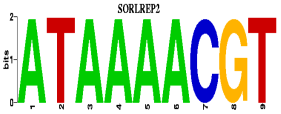

Sequence logo generation powered by weblogo
STAMP is written by Shaun Mahony
