# Supplementary material for: Tinkering Cis Motifs Jigsaw Puzzle Led to Root-Specific Drought-Inducible Novel Synthetic Promoters
Source: Int J Mol Sci. 2020 Feb 18;21(4):1357. doi: 10.3390/ijms21041357 (PMC7072871; doi:10.3390/ijms21041357)
Supplement: Supplementary file 1 [file ijms-21-01357-s001.zip › Supplementary Files/Table S1-List of upregulated genes.docx]

**Table S1. List of 63 genes upregulated in soybean under drought stress**

| **S No.** | **Gene ID** |
| --- | --- |
| 1 | Glyma01g02110 |
| 2 | Glyma01g07390 |
| 3 | Glyma01g24530 |
| 4 | Glyma01g32090 |
| 5 | Glyma01g41330 |
| 6 | Glyma01g44930 |
| 7 | Glyma02g13120 |
| 8 | Glyma02g40800 |
| 9 | Glyma02g41070 |
| 10 | Glyma02g47380 |
| 11 | Glyma03g37140 |
| 12 | Glyma04g02330 |
| 13 | Glyma04g04170 |
| 14 | Glyma04g06520 |
| 15 | Glyma04g37040 |
| 16 | Glyma04g40960 |
| 17 | Glyma05g08950 |
| 18 | Glyma05g35830 |
| 19 | Glyma06g02380 |
| 20 | Glyma06g06550 |
| 21 | Glyma06g15990 |
| 22 | Glyma06g22210 |
| 23 | Glyma06g42180 |
| 24 | Glyma07g04860 |
| 25 | Glyma07g05800 |
| 26 | Glyma08g03780 |
| 27 | Glyma08g03850 |
| 28 | Glyma08g06110 |
| 29 | Glyma08g06440 |
| 30 | Glyma08g09450 |
| 31 | Glyma09g26080 |
| 32 | Glyma09g29840 |
| 33 | Glyma10g00430 |
| 34 | Glyma10g11000 |
| 35 | Glyma10g28290 |
| 36 | Glyma10g36200 |
| 37 | Glyma11g05960 |
| 38 | Glyma11g16070 |
| 39 | Glyma11g34410 |
| 40 | Glyma12g10670 |
| 41 | Glyma12g35000 |
| 42 | Glyma13g29270 |
| 43 | Glyma13g35550 |
| 44 | Glyma14g32430 |
| 45 | Glyma14g36860 |
| 46 | Glyma15g07930 |
| 47 | Glyma15g14290 |
| 48 | Glyma15g40070 |
| 49 | Glyma16g02390 |
| 50 | Glyma17g09850 |
| 51 | Glyma17g13720 |
| 52 | Glyma18g01720 |
| 53 | Glyma18g03930 |
| 54 | Glyma18g04020 |
| 55 | Glyma18g06840 |
| 56 | Glyma18g07970 |
| 57 | Glyma18g50240 |
| 58 | Glyma19g02710 |
| 59 | Glyma19g11770 |
| 60 | Glyma19g28550 |
| 61 | Glyma19g39950 |
| 62 | Glyma19g40550 |
| 63 | Glyma20g22700 |
